# Characterization of a novel AraC/XylS-regulated family of N-acyltransferases in pathogens of the order Enterobacterales

**DOI:** 10.1371/journal.ppat.1008776

**Published:** 2020-08-26

**Authors:** Laura Belmont-Monroy, Waleska Saitz-Rojas, Jorge Soria-Bustos, Abigail S. Mickey, Nicholas E. Sherman, Benjamin C. Orsburn, Fernando Ruiz-Perez, Araceli E. Santiago

**Affiliations:** 1 Department of Pediatrics, University of Virginia School of Medicine and University of Virginia Children’s Hospital, Charlottesville, Virginia, United States of America; 2 Department of Public Health, UNAM School of Medicine and Federico Gomez Children’s Hospital, Mexico City, Mexico; 3 W. M. Keck Biomedical Mass Spectrometry Lab. University of Virginia, Charlottesville, Virginia, United States of America; University of Utah, UNITED STATES

## Abstract

Enteroaggregative *Escherichia coli* (EAEC) is a diarrheagenic pathotype associated with traveler’s diarrhea, foodborne outbreaks and sporadic diarrhea in industrialized and developing countries. Regulation of virulence in EAEC is mediated by AggR and its negative regulator Aar. Together, they control the expression of at least 210 genes. On the other hand, we observed that about one third of Aar-regulated genes are related to metabolism and transport. In this study we show the AggR/Aar duo controls the metabolism of lipids. Accordingly, we show that AatD, encoded in the AggR-regulated *aat* operon (*aatPABCD*) is an N-acyltransferase structurally similar to the essential Apolipoprotein N-acyltransferase Lnt and is required for the acylation of Aap (anti-aggregation protein). Deletion of *aatD* impairs post-translational modification of Aap and causes its accumulation in the bacterial periplasm. *trans-*complementation of 042*aatD* mutant with the AatD homolog of ETEC or with the N-acyltransferase Lnt reestablished translocation of Aap. Site-directed mutagenesis of the E207 residue in the putative acyltransferase catalytic triad disrupted the activity of AatD and caused accumulation of Aap in the periplasm due to reduced translocation of Aap at the bacterial surface. Furthermore, Mass spectroscopy revealed that Aap is acylated in a putative lipobox at the N-terminal of the mature protein, implying that Aap is a lipoprotein. Lastly, deletion of *aatD* impairs bacterial colonization of the streptomycin-treated mouse model. Our findings unveiled a novel N-acyltransferase family associated with bacterial virulence, and that is tightly regulated by AraC/XylS regulators in the order Enterobacterales.

## Author summary

Although the regulatory scheme of AggR is well understood, the biological relevance of half of AggR-regulated proteins remains unknown. In this study we provide experimental evidence that the AggR-regulated AatD is a novel N-acyltransferase restricted to pathogens of the order Enterobacterales, including EAEC, ETEC, *Yersinia* sp., and *C*. *rodentium*. AatD is structurally similar to Lnt. However, unlike Lnt which is essential for cellular functions, AatD is a dedicated N-acyltransferase required for post-translational modification of virulence factors. Aap was identified as a lipoprotein acylated by AatD. Lipid modification in Aap provides an important post-translational mechanism to regulate the trafficking, stability and subcellular localization of Aap. In the absence of AatD, Aap is retained in the periplasmic space and cannot be translocated to the bacterial surface, presumably, restricting the biological function of the protein. Our data suggest that AggR and Aar virulence regulators, not only regulate the expression of Aap virulence factor at the transcriptional level, but also regulate translocation of Aap to the bacterial surface, which is required for full virulence of EAEC, unveiling an important mechanism of virulence regulation.

## Introduction

Enteroaggregative *Escherichia coli* (EAEC) is a diarrheagenic pathotype linked to traveler’s diarrhea, foodborne outbreaks and sporadic diarrhea in industrialized and developing countries [1–7]. Virulence gene expression in EAEC is activated in coordinated fashion by a regulator called AggR, a member of the AraC/XylS family of bacterial transcription factors [8–10]. AggR in EAEC042 regulates the expression of at least 44 genes located in the pAA virulence plasmid and bacterial chromosome including the genes encoding AAF (Aggregative Adherence Fimbriae), *aap* (dispersin surface protein), the AAT operon (a Type-I Secretion System) required for transport of dispersin to the bacterial surface, genes encoding the Type VI secretion system and its own negative regulator Aar (AggR activated regulator) [8]. Aar, a member of ANR (AraC Negative Regulator) family, binds directly to AggR, thus inhibiting the latter’s ability to bind to bacterial promoter regions [11].

Although the regulatory scheme of AggR is well understood, little is known about whether AggR-regulated virulence factors are further processed to be fully functional. Pathogens use various mechanisms to manipulate the availability or the function of virulence factors by posttranslational modifications that alter the activity, localization, or interactions of the modified protein. One of these posttranslational modifications is acylation. The most common form of acylation in *E*. *coli* is myristoylation and palmitoylation, where proteins are modified with myristate (14-carbon) or palmitate (16-carbon) saturated fatty acids, respectively. Myristoylated proteins have a myristate linked to Gly-2 via an amide, whereas palmitoylated proteins contain palmitate linked to the N-terminal (N-acylation) or the sulfhydryl group (S-acylation) of cysteine [12–14]. The Bacteroidales produce an N-acylated derivative of glycine, which is another subclass of the N-fatty acyl amino acids [15].

Lipoproteins are synthesized in the bacterial cytoplasm and translocated across the inner membrane via the Tat or Sec apparatus and post-translationally modified in the inner membrane [14]. Lipoproteins contain an N-terminal signal peptide (also known as the lipobox) with a cysteine residue targeted for lipid modification. Lipid attachment occurs via a sequential 3-step process, in the first step the cysteine receives an 1,2-diacylglyceryl group from phosphatidylglycerol through the action of diacylglyceryl transferase (Lgt) [13, 14]. The amino-terminal signal peptide is then processed by prolipoprotein signal peptidase (LspA). In the last step, N-acyltransferase (Lnt) catalyzes the transfer of an acyl chain from a phospholipid to the amine group of the N-terminal cysteine residue of the apolipoprotein [12, 16–18]. Lnt in *E*. *coli* can use all available phospholipids, but phosphatidylglycerol is the preferred substrate [12, 13]. Finally, LolABCDE, an ABC transporter system, release the mature lipoproteins in the outer membrane (OM) [19–21]. N-acylation of apolipoproteins by Lnt is necessary for efficient recognition of outer membrane lipoproteins by the Lol system [22, 23]. Lnt deletion in *E*. *coli* is lethal partially because of the retention of apoLpp in the plasma membrane [22].

Here, we provide experimental evidence that AatD, a component of the *aat* operon is a tightly regulated N-acyltransferase associated with virulence and restricted to pathogens of the order Enterobacterales, and show that at least one of its functions is to regulate the trafficking and location of Aap.

## Results

### Aar controls the metabolism of lipids in EAEC

**Fig 1:**
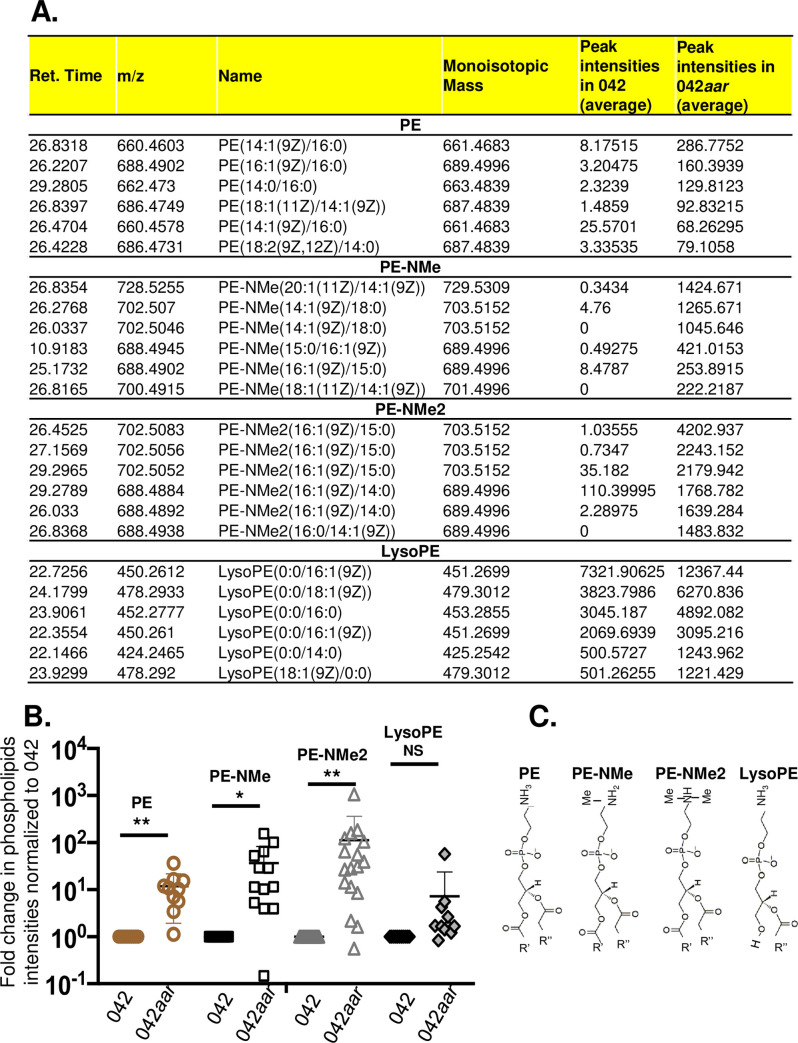
Untargeted metabolomic analysis (UMA) of 042 and 042*aar*. EAEC 042 and 042*aar* derivatives were grown in DMEM high glucose for 6 h and processed for UMA by Liquid chromatography / mass spectrometry (LC/MS). Differential amounts of phosphatidylethanolamine (PE, PE-NMe, PE-NMe2) and lysophosphatidylethanolamine (LysoPE) lipid species were detected between these strains. The averaged intensities from 2 samples run in duplicates for six of the most abundant PE and LysoPE species are shown in Panel A (whole raw data for remaining species are shown in S1A and S1B Table). Unpaired T-test statistical analysis of individual PE and Lyso-PE data points normalized to wild type 042 from two independent experiments run in duplicate are shown in panel B (*, P < 0.01; **, P < 0.001). General lipid structure of phosphatidylethanolamine species (PE) and Lysophosphatidylethanolamine (LysoPE) species identified in the UMA are depicted in panel C. UMA raw data were acquired and aligned by using the Makerlynx software (version 4.1) based on the m/z value and the retention time of ion signals. Metabolites were identified using the iMass Bank database.

In EAEC, regulation of virulence is mediated by AggR and its negative regulator Aar. Aar controls the expression of at least 210 genes; one-third of these genes are related to metabolism and transport [24]. We sought to determine the relevance of AggR/Aar-mediated regulation of metabolic pathways in EAEC by performing untargeted metabolomic analysis of EAEC042 and EAEC042*aar*. Briefly, strains were grown in DMEM high glucose and bacterial pellets were analyzed by Liquid chromatography–mass spectrometry **(**LC/MS) (Creative Proteomics Inc, New Jersey). Although many metabolic products were detected, our data consistently showed differential amount of phosphatidylethanolamine (PE) and lysophosphatidylethanolamine (LysoPE) lipid species between these strains as judged by metabolomic analysis of two independent experiments run in duplicates, suggesting that AggR/Aar are involved in the regulation of lipid metabolism (Fig 1A–1C and S1A and S1B Table).

Since PE and LysoPE species serve as the donor of fatty acids in the N-acylation of apolipoproteins generating lysophospholipids as products of degradation by action of N-acyltransferases, we sought to identify AggR/Aar-regulated proteins with N-acyltransferase activity. With this purpose, we took advantage of previously reported microarray and RNAseq data from EAEC 042*aggR* and EAEC 042*aar* strains [8, 24], which identified one potential hypothetical N-acyltransferase; AatD (CBG27765.1, EC042_pAA011) [8, 25]. AatD is encoded in the *aat* operon and its function remains obscure [25].

### *In-silico* analysis suggests that AatD belongs to a novel family of N-acyltransferases in pathogens of the order Enterobacterales

AatD homologs were retrieved from the NCBI BLAST web server and analyzed by PROMALS3D algorithm (S1 Fig) [26, 27]. PROMALS3D algorithm strongly predicted the presence of highly conserved αββα structural domains in the Glu(E)-Lys(K)-Cys(C) catalytic triad region (~200–400 aa) of members of the family (S1 Fig).

**Fig 2:**
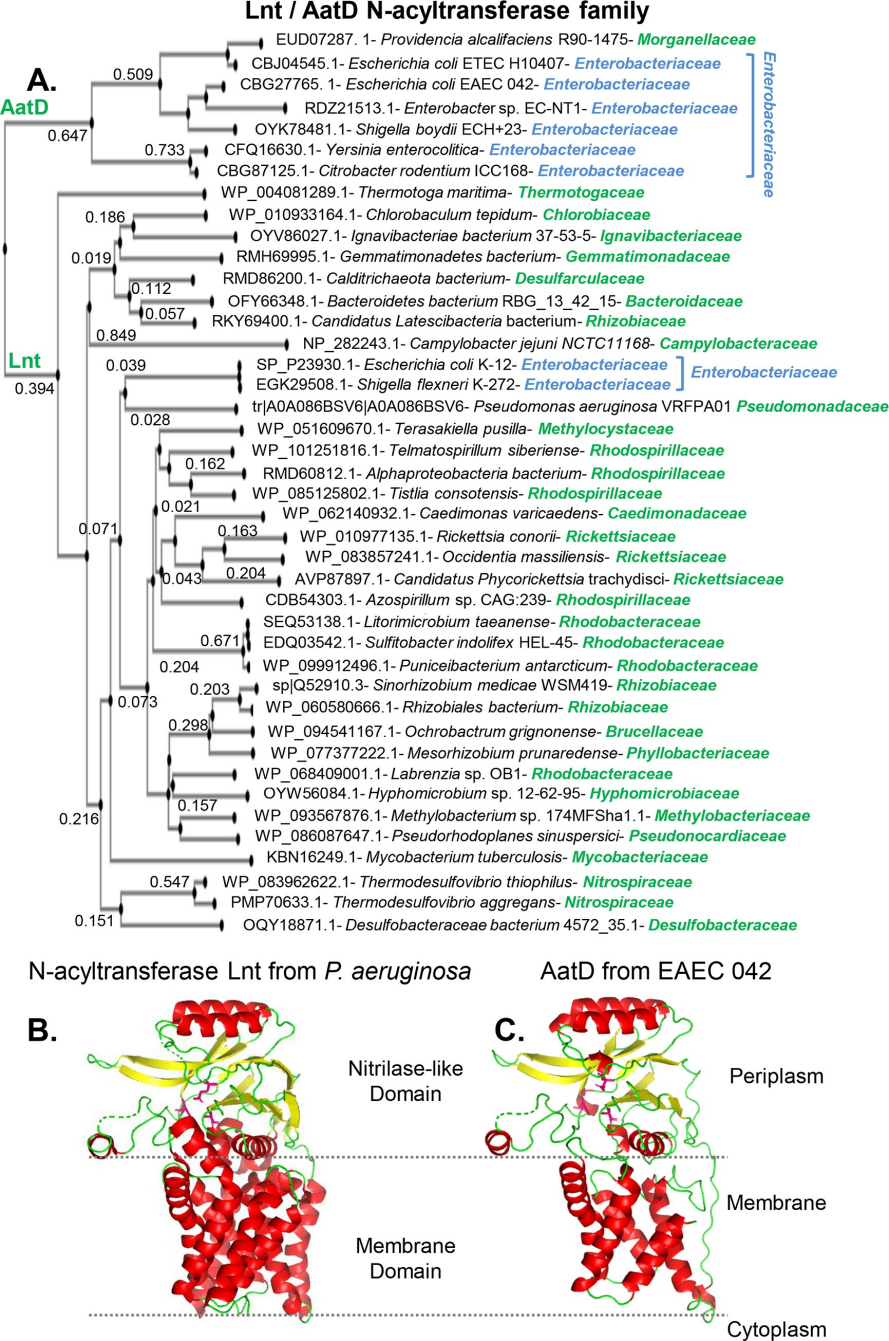
Phylogenetic analysis of AatD / Lnt family. Phylogenetic analysis of the aminoacid sequence of Lnt-AatD family using the MAFFT algorithm, subdivided the family into two well-conserved N-acyltransferase lineages; those close to AatD or to Lnt (Panel A). The AatD was only found in pathogens of the order Enterobacterales (annotated in blue). A hypothetical structural model for AatD was generated based on its closest structural homolog Lnt from *P*. *aeruginosa* (5N6M) by using Phyre2 algorithm, with 99.2% of confidence (Panels B and C).

Phylogenetic analysis of the amino acid sequence of these N-acyltransferases using the MAFFT algorithm [28], subdivided the family into two well-conserved lineages; those close to AatD or to Lnt (Fig 2A). AatD homologs were identified in pathogenic bacteria of the order Enterobacterales including *E*. *coli* (EAEC and ETEC), *Shigella boydii*, *Yersinia enterocolitica*, *Enterobacter* sp, and *Citrobacter rodentium* (Fig 2A). AatD was also found in *Providencia alcalifaciens*, an opportunist member of *Morganellaceae* family that is associated with gastroenteritis in humans [29]. In contrast to EAEC and ETEC, which harbor *aatD* in a virulence plasmid, the homologous *aatD* gene of *C*. *rodentium* was located at the bacterial chromosome. Lnt homologs were distributed among more than 20 bacterial families including *Enterobacteriaceae*, *Rhizobiaceae*, *Brucellaceae*, *Rhodospirillaceae*, *Rickettsiaceae*, *Pseudomonadaceae* and *Bacteroidaceae* (Fig 2A).

A hypothetical structural model for AatD was generated based on its closest structural homolog Lnt from *P*. *aeruginosa* (5N6M) using the algorithm Phyre2 [30, 31], in which 378 residues of AatD (94% of AatD sequence) matched to the single highest scoring template corresponding to Lnt (5N6M) with 99.2% confidence (Fig 2B and 2C).

### Aap accumulates in the periplasm of 042*aatD*

It has been shown that deletion of *lnt* results in the accumulation of apolipoproteins in the periplasm [22, 32]. It was shown that deletion of gene components of the *aat* operon (including *aatD)* causes retention of Aap in the bacterial periplasm [25]. Retention of Aap in EAEC bearing a non-functional Aat translocator was easily comprehended, but retention of Aap in the absence of AatD, which does not form part of the Aat translocator itself, was puzzling. To further explore the significance of this phenotype given the resemblance of AatD with Lnt, we reproduced the initial findings by analyzing the presence of Aap in the periplasmic fractions of Wt 042, 042*aatA*, 042*aatC* and 042*aatD* (S2 Fig). Interestingly, we observed that Aap accumulates more abundantly in the *aatD* mutant as judged by blue coomassie staining and western blot analysis (S2 Fig).

**Fig 3:**
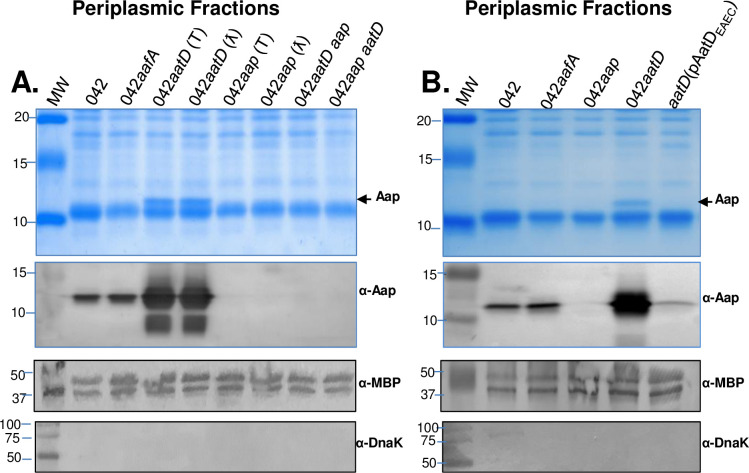
Aap accumulates in the periplasm of 042*aatD*. Periplasmic fractions of 042 derivatives containing individual deletions of *aafA*, *aap*, *and aatD* or double deletion (042*aatD/aap* and 042*aap/aatD*); created by transposon (T) or lambda red (λ) mutagenesis procedures, were analyzed by Coomassie blue in 20% SDS-PAGE and Western blot using a polyclonal antibody against Aap (Panel A). 042*aatD aap* and 042*aap aatD* strains have deletions in *aap* and *aatD* but the Km marker is found in the *aap* or *aatD* locus, respectively. The 042*aatD* strain was complemented *in trans* with pAatD_EAEC_ plasmid and analyzed as in Panel B. Anti-MBP and anti-DnaK antibodies were used as markers of periplasmic and cytoplasmic fractions, respectively.

To rule out that this was not due to an artifact, 042 derivatives containing single non-polar deletions in *aap* and *aatD* or double deletions in *aap* and *aatD* were generated by two different techniques; lambda-red (λ) and transposon (T) mutagenesis. 042 derivatives were grown overnight in DMEM-HG. As control, a 042 mutant in *aafA* (major fimbrial subunit of aggregative adherence fimbria II, AAF/II) was included. Periplasmic preps were obtained and analyzed by SDS-PAGE and Western blot (WB) (Fig 3A). The protein profile in the gel stained with coomassie blue was similar between the strains, with the exception of an extra ~12 kDa band easily detected in 042*aatD* mutants by the naked eye (Fig 3A and 3B). The band corresponded to Aap since it was absent in all *aap* mutants as confirmed by WB (Fig 3A and 3B). Low amounts of periplasmic Aap were seen in the Wt 042 and 042*aafA*. As expected, Aap was not present at the periplasmic fraction of 042*aap*, 042*aap aatD* (this strain has a Km marker inserted in the *aatD* locus) and 042*aatD aap* (this strain has a Km marker inserted in the *aap* locus) mutants. Complementation *in trans* of 042*aatD* with a plasmid expressing AatD restored the WT protein profile (Fig 3B). Our experimental findings confirm that in the absence of AatD, Aap accumulates in the periplasm of 042*aatD*.

### Abundance and trafficking of Aap in absence of AatD

**Fig 4:**
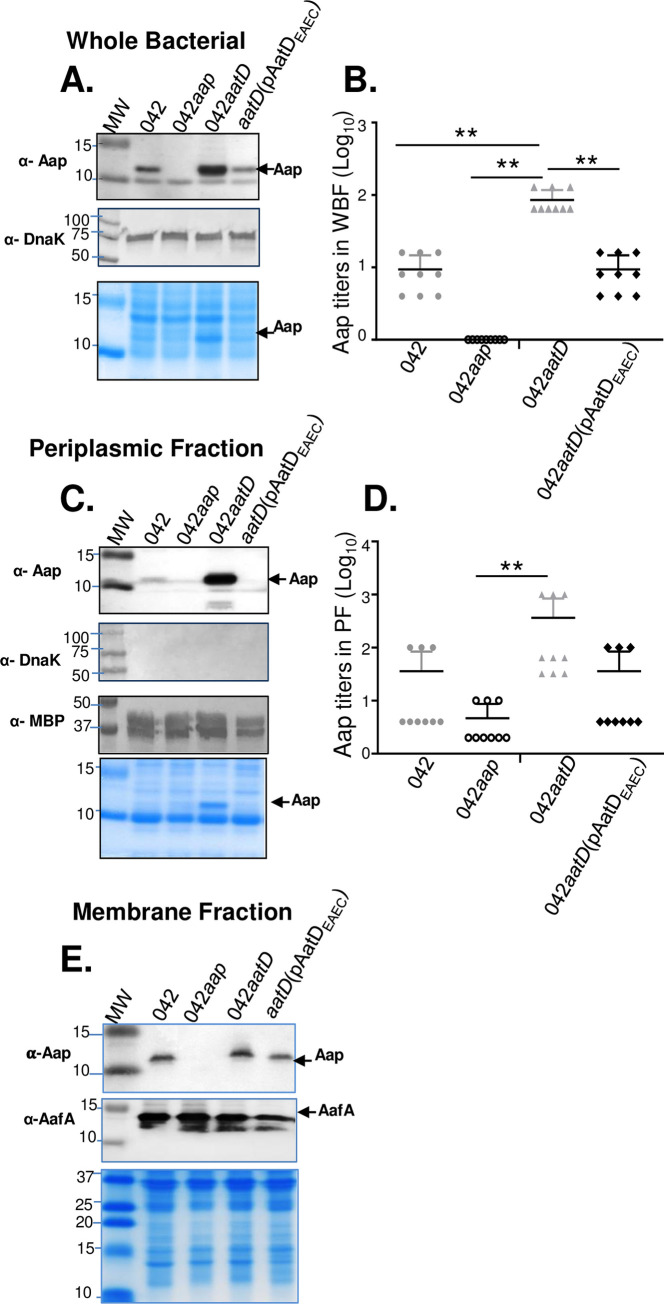
Compartmentalization of Aap is affected in absence of AatD. Aap was analyzed in whole bacterial fractions (WBF) (Panels A and B), periplasmic fractions (PF) (Panels C and D) and membrane fractions (MF) (Panel E) of 042 derivatives by Coomassie blue SDS-PAGE and Western blot. Relative amounts of Aap were quantified in WBF and PF by ELISA using a polyclonal antibody against Aap (Panels B and D). Data are representative of three independent experiments run in triplicates. Anti-MBP and Anti-DnaK antibodies were used as markers of periplasmic and cytoplasmic fractions, respectively. Anti-AafA antibody was used as a marker for MF. Asterisks indicate significant difference by one-way ANOVA Bonferroni’s post-test (**, P < 0.001).

It is well documented that depletion of the N-acyltransferase Lnt causes mislocalization of outer membrane lipoproteins in *E*. *coli* [22, 32]. Since dispersin has been localized in the outer membrane [10, 25, 33], we sought to determine more extensively how the absence of *aatD* affects trafficking of Aap in the 042 strain. For these experiments, whole bacterial fractions (WBF), periplasmic fractions (PF) and membrane fractions (MF) were obtained and analyzed by SDS-PAGE and WB (Fig 4A, 4C and 4E). Unexpectedly, Aap was not only found in reduced amounts in PF, but also in WBF of Wt 042 when compared with isogenic 042*aatD* mutant. Aap was ~10-fold higher in WBF and PF fractions of 042*aatD* than Wt 042 (Fig 4B and 4D). Nevertheless, Aap was found at similar levels in the MF of both strains (Fig 4E).

**Fig 5:**
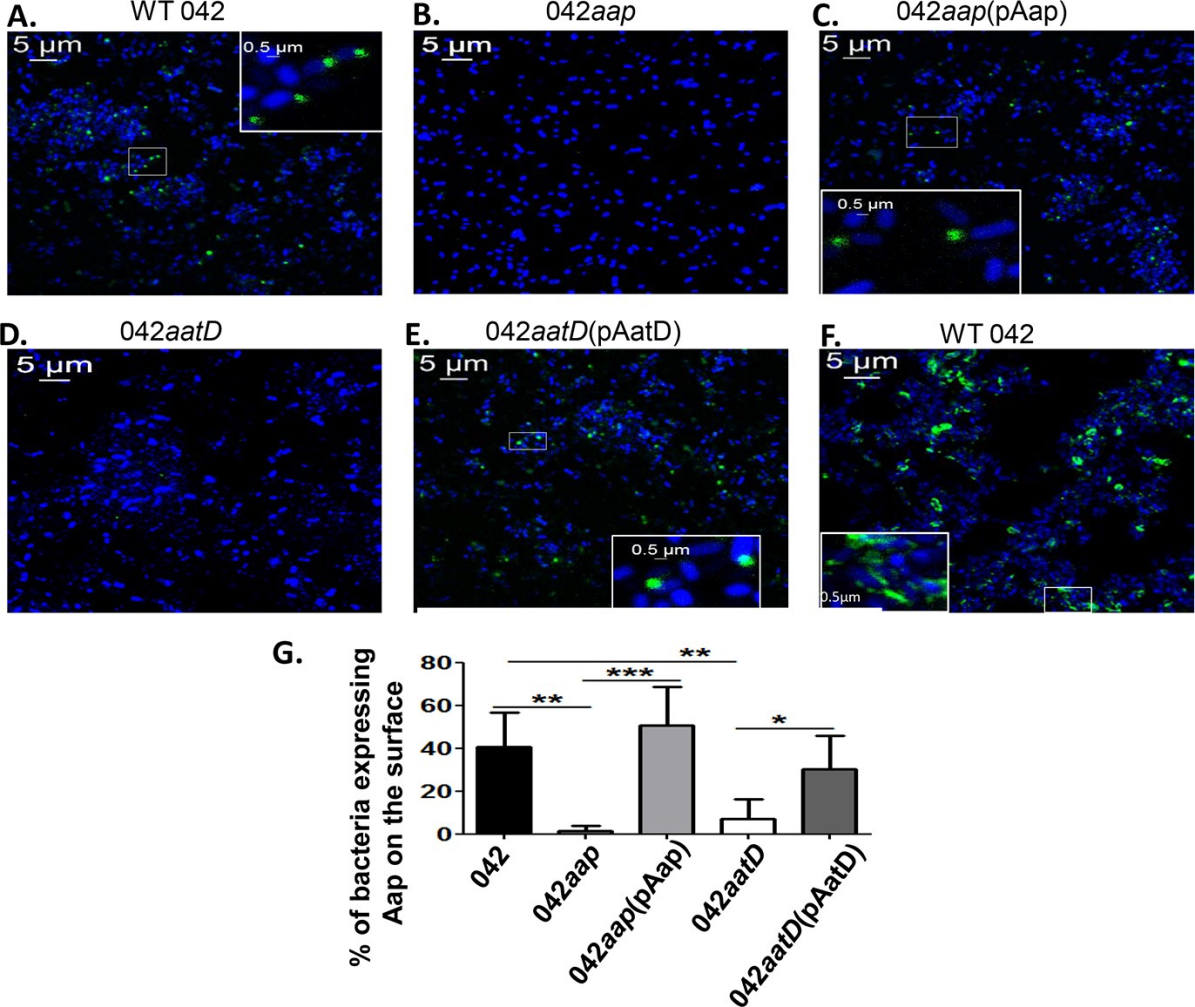
AatD is required for efficient translocation of Aap to the bacterial surface. 042 derivatives were grown statically in DMEM overnight at 37°C. Bacterial cells were harvested, washed with PBS and incubated with anti-Aap polyclonal antibody for 1 h, followed by incubation with Alexa-488-conjugated secondary antibody (green, for Aap staining) and Hoechst 33342 stain (blue, for DNA staining) (Panel A-E). As a control for surface localization, Wt 042 was incubated with anti-AafA polyclonal antibody and Alexa-488-conjugated secondary antibody (green, for fimbriae staining) (Panel F). Bacterial cells were analyzed using a LSM-710 laser-scanning confocal microscope (Zeiss, Germany). Representative confocal images taken with the 64X oil objective are shown (Panel A-F). Polar localization of Aap at the bacterial surface is shown in magnified sections of the images (Panel A, C, E and F). Percentage of bacteria expressing Aap on the surface from the total bacteria population of 5–6 microscopic fields, of at least three independent experiments, is shown in panel G. Asterisks indicate significant difference by one-way ANOVA Bonferroni’s post-test (*, P < 0.05; **, P < 0.001; ***, P < 0.0001).

We therefore analyzed whether Aap was translocated at the bacterial surface in the *aatD* mutant by immunofluorescence using a polyclonal antibody against Aap (Fig 5). Interestingly, at difference of AafA fimbriae (positive control) that was distributed around the bacteria (Fig 5F), Aap was found mostly localized at the bacterial poles in strains with functional AatD (Fig 5A, 5C and 5E). We also found that not all strains having a functional AatD exhibited Aap on the bacterial surface, as only approximately 35–50% of the whole bacterial population were stained with the anti-Aap polyclonal antibody (Fig 5G). We did not observe Aap on the surface of 042*aap* and 042*aatD* strains (Fig 5B, 5D and 5G). Taken together, these studies provide experimental evidence that AatD is required for efficient translocation of Aap to the bacterial surface.

### Aap accumulates at the poles in the periplasm of 042*aatD*

**Fig 6:**
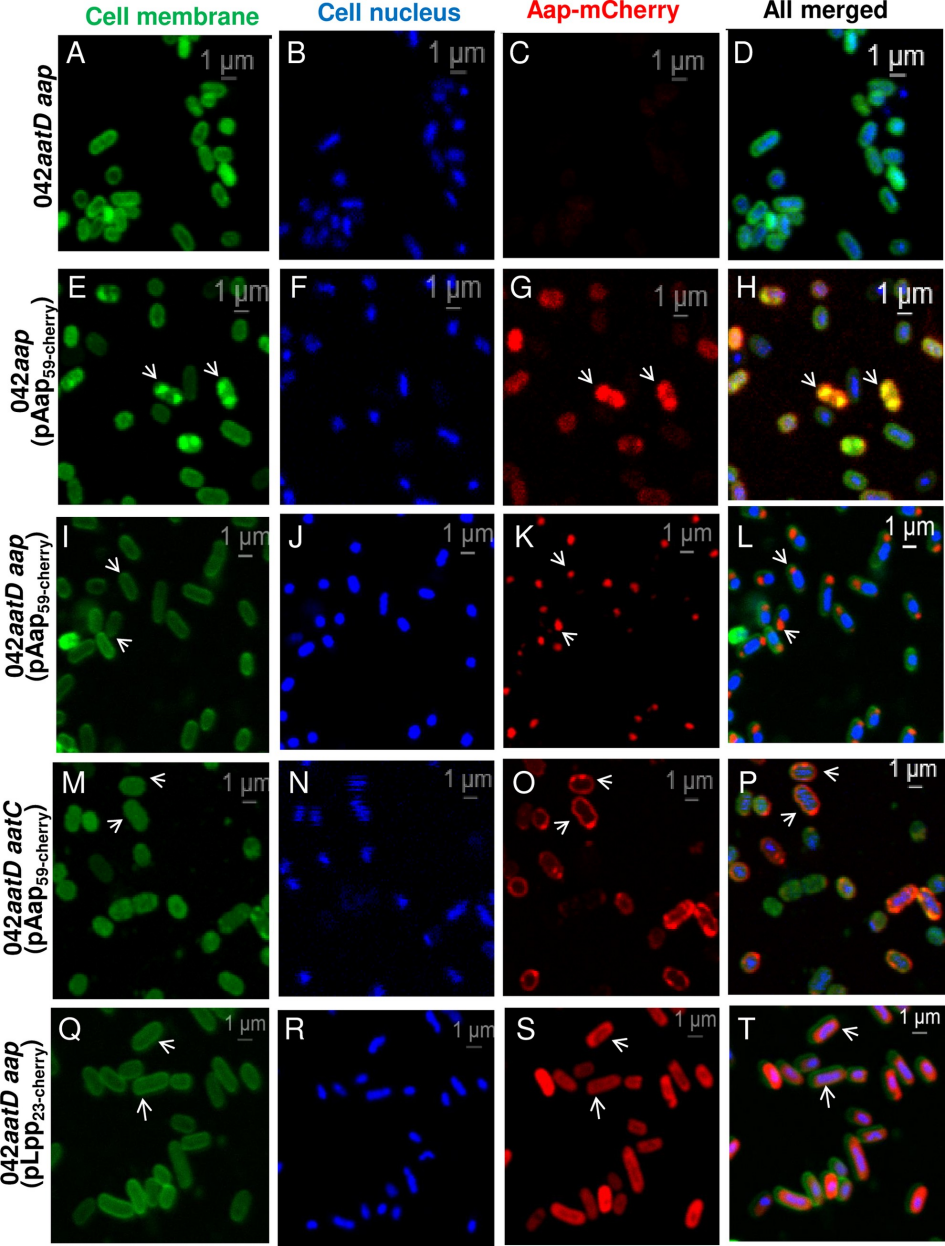
Aap-mCherry accumulates at the poles in the periplasm of 042*aatD*. 042 and 042 derivatives (042*aap*, 042*aatD aap* and 042*aatD aatC)* transformed with pAap_59-cherry_ were grown statically in DMEM overnight at 37°C. Bacterial cells were harvested, washed with PBS and incubated with CellBrite stain (green, for membrane staining) and Hoechst 33342 stain (blue, for DNA staining) for 1 h. Bacterial cells were analyzed using a LSM-710 laser-scanning confocal microscope (Zeiss, Germany). Magnified sections of representative confocal images taken with the 64X oil objective are shown (Panel A-T). The localization of Aap_59-cherry_ overlapping with bacterial membranes (Panel G, H), at the periplasmic poles (Panel K, L) and homogenously diffused in the periplasm (Panel O, P) are indicted with arrows. 042*aatD aap* (pLpp_23-cherry_) was included as a control (S, T). Original images are shown in S3 Fig

Although the precise biological role of Aap remains unknown, its compartmentalization might be crucial for its function. Since we observed polar localization of native Aap at the bacterial surface of Wt 042 and accumulation of Aap in the periplasm of 042*aatD*, we sought to visualize the compartmentalization of Aap in 042 and 042*aatD* strains using an alternative approach. For these experiments, the first 59 amino acids of Aap were fused to the reporter mCherry (Aap_59-cherry_) and expressed under the constitutive P*aar* promoter [34]. 042 derivatives 042*aap*, 042*aatD aap* and 042*aatD aatC* were transformed with the plasmid pAap_59-cherry_ and analyzed by confocal microscopy (Fig 6 and S3 Fig). The latter strain also lacks of the ATPase AatC, the provider of energy necessary for a functional Aat translocator [25]. As a control we used the pLpp_23-cherry_ plasmid encoding the first 23 amino acids of the well-characterized lipoprotein Lpp fused to mCherry, and which has been successfully used to study the trafficking of lipoproteins in the LolABCDE transporter system [35].

We found that Aap_59-cherry_ is retained in the periplasmic space and accumulates at the poles of 042*aatD aap* (Fig 6I–6L). However, in the presence of AatD, Aap_59-cherry_ mostly colocalized with the bacterial membrane as judged by the overlapping of Aap_59-cherry_ and CellBrite (which stain membranes), visible as a yellow color in the merged image (Fig 6E–6H). Polar localization of Aap_59-cherry_ was not an artifact due to the fusion with mCherry since Lpp_23-cherry_ does not localizes at the bacterial poles (Fig 6Q–6T). Of note, in the absence of the ATPase AatC, Aap_59-cherry_ appears more diffused in the periplasm, with few accumulations at the poles of 042*aatD aatC* mutant (Fig 6M–6P). Taken together, these results suggest that the native Aap is routed out to the poles where is translocated to the bacterial outer membrane.

### Mutation of E207 residue in the AatD catalytic triad impairs translocation of Aap

**Fig 7:**
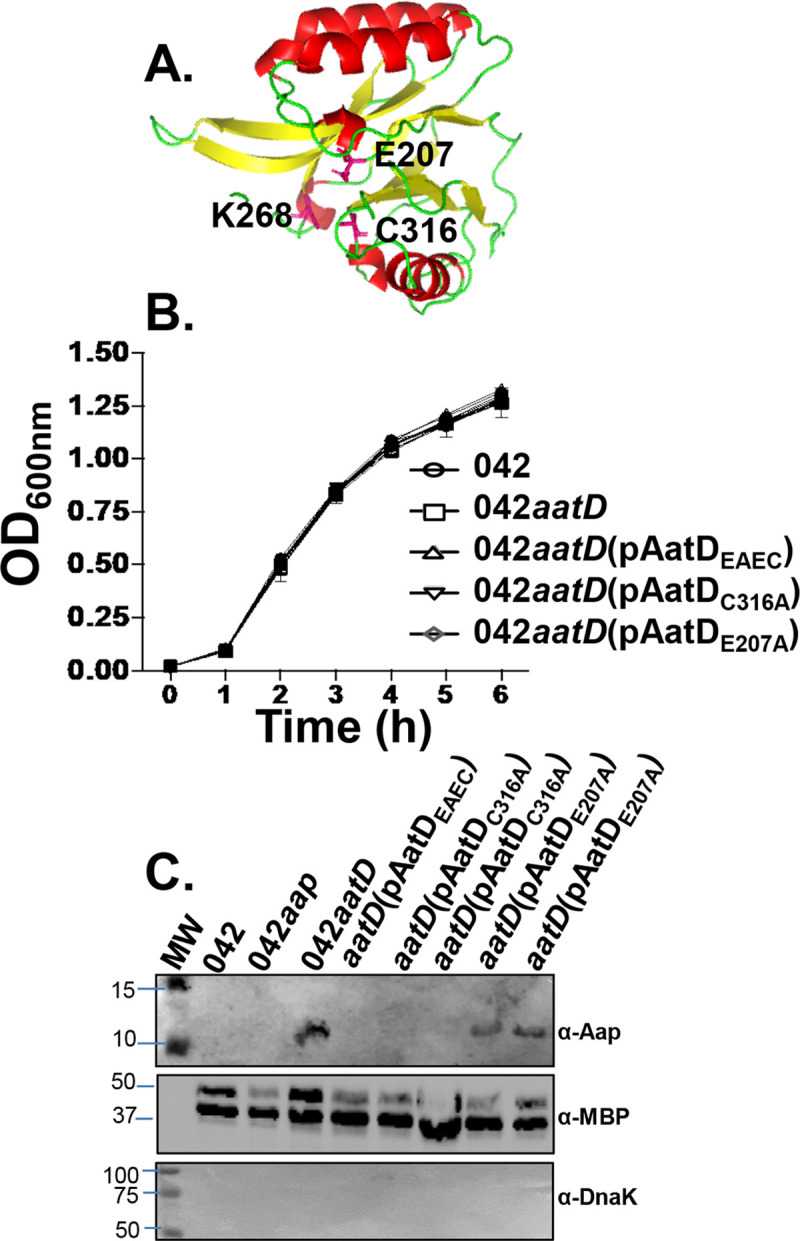
Mutation of E207 residue in the AatD catalytic triad causes accumulation of Aap in the periplasm. Residues E207 and C316 in the putative catalytic triad of AatD (illustrated in Panel A) were changed to A (Ala) by site-directed mutagenesis. The grow rates of the 042*aatD* strain transformed with plasmids encoding AatD variants (pAatD_E207A_, pAatD_C316A_ and pAatD_EAEC_), Wt 042 and 042*aatD* were assessed in triplicate at 37°C over 6 h period (Panel B). Periplasmic fractions of these strains were analyzed by western blot using anti-Aap, anti-MBP and anti-DnaK antibodies (Panel C).

We sought to determine whether mutations in the putative acyltransferase catalytic triad of AatD may affect the function of AatD (Fig 7). Accordingly, site-directed mutagenesis was performed on the three residues (E207, K268 and C316) conforming the putative catalytic triad of AatD (Fig 7A). We found that mutation of K268 affected the viability of 042, while mutation of E207 or C316 did not cause detrimental effects in 042 (Fig 7B). Mutation of C316 did not show drastic effects in AatD function, but mutation of E207 residue affected the function of AatD as judged by accumulation of Aap in the periplasm (Fig 7C) and reduction in the expression of Aap on the bacterial surface. Our data suggest that the absence of AatD or alteration of its putative N-acyltransferase catalytic triad affects the trafficking of Aap.

### EAEC 042*aatD* can be complemented in *trans* by Lnt

**Fig 8:**
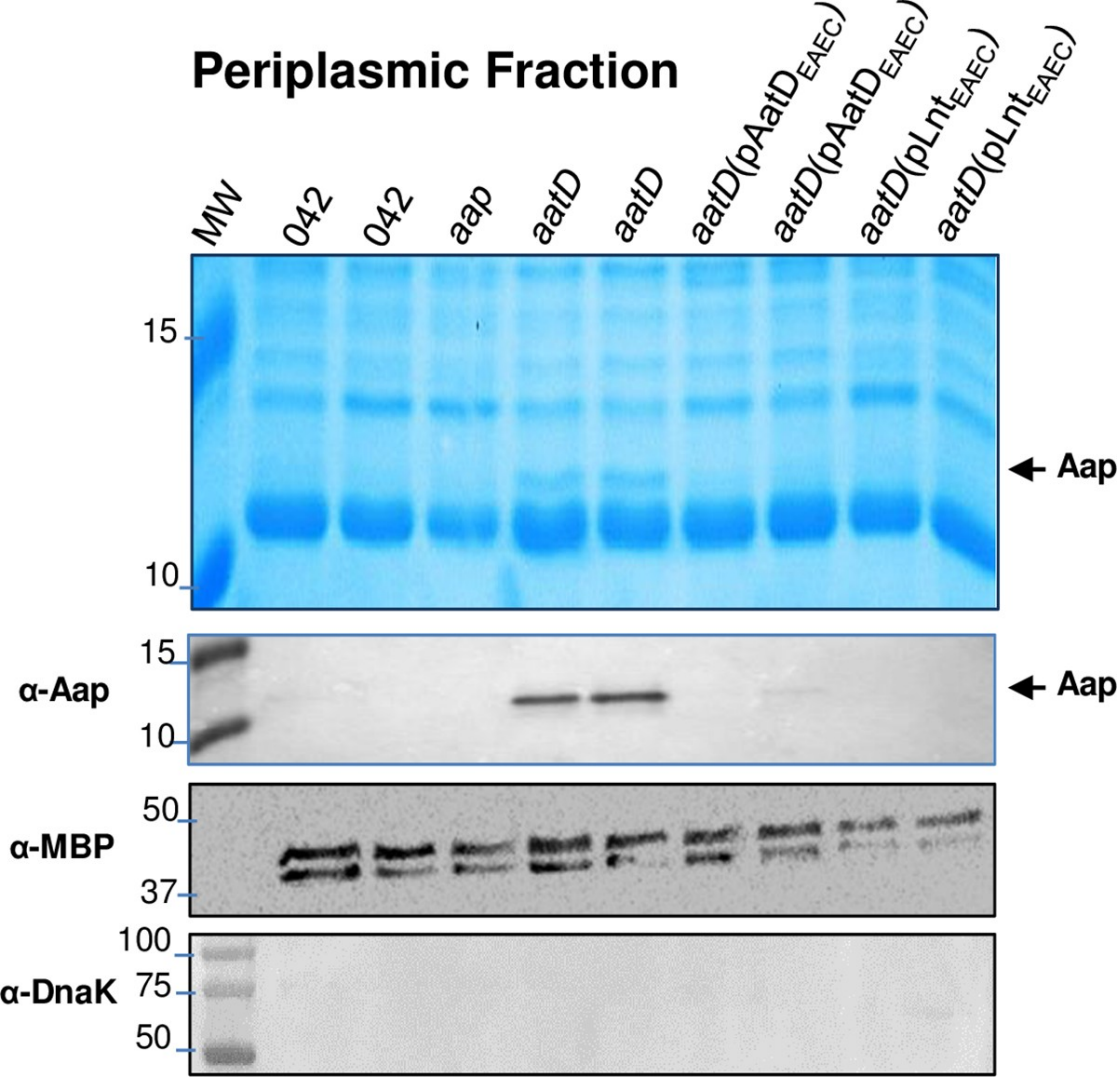
Aap does not accumulate in the periplasm of 042*aatD* complemented with Lnt. 042*aatD* strain was transformed with pLnt_EAEC_ and control pAatD_EAEC_ plasmid. Periplasmic fractions of 042*aatD* derivatives were analyzed by coomassie blue stain SDS-PAGE and western blot using anti-Aap polyclonal antibody. Anti-MBP and anti-DnaK antibodies were used as markers of periplasmic and cytoplasmic fractions, respectively.

**Fig 9:**
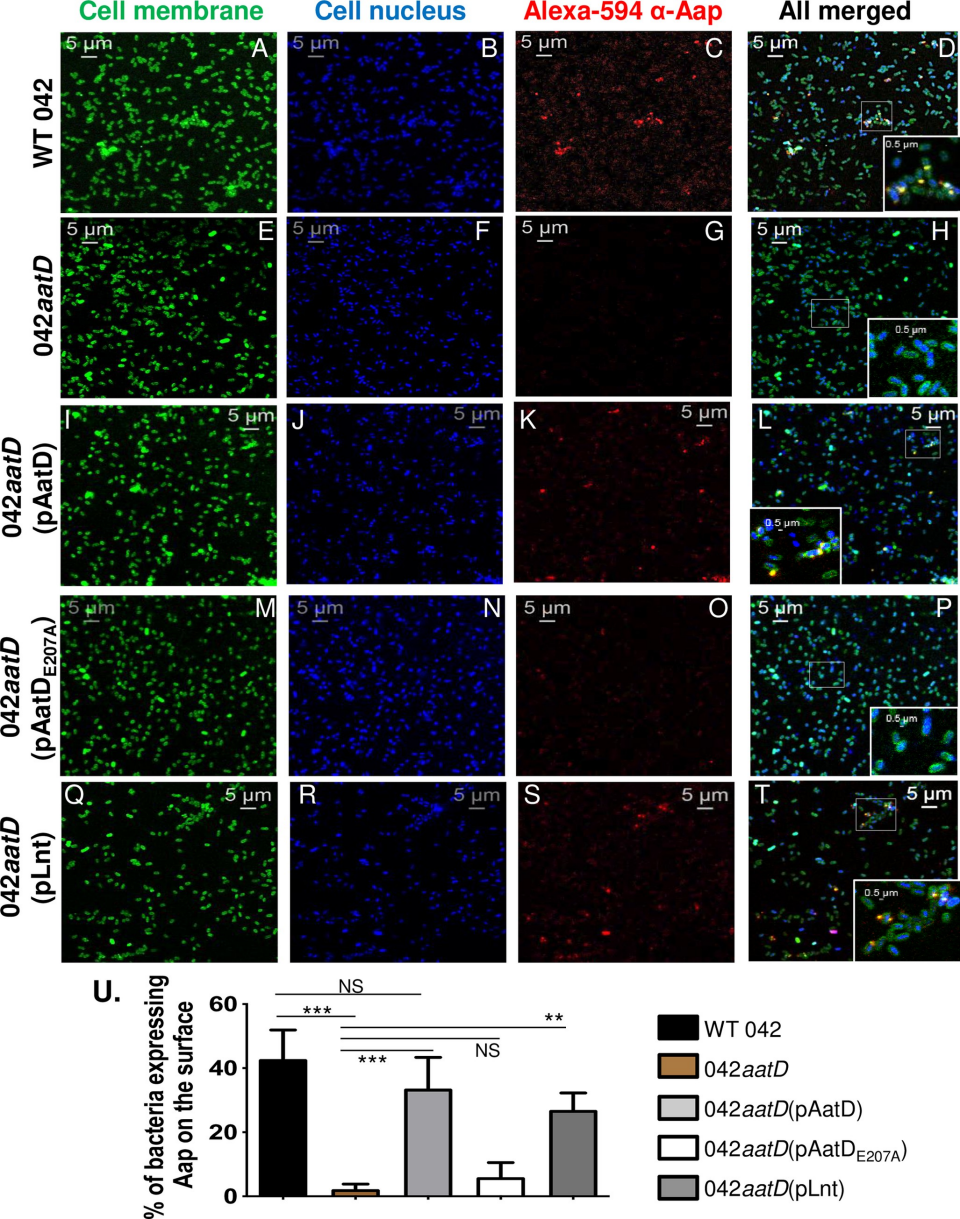
AatD and Lnt, but not AatD_E207A_ can reestablish surface-expression of Aap in 042*aatD*. 042 derivatives were grown statically in DMEM overnight at 37°C. Bacterial cells were harvested, washed with PBS and incubated with anti-Aap polyclonal antibody for 1 h, followed by incubation with Alexa-594-conjugated secondary antibody (red, for Aap staining), CellBrite (green, for bacterial membrane staining) and Hoechst 33342 (blue, for DNA staining). Bacterial cells were analyzed using a LSM-710 laser-scanning confocal microscope (Zeiss, Germany). Representative confocal images taken with the 64X oil objective are shown (Panel A-T). Polar localization of Aap overlapping with membranes at the bacterial surface is shown in magnified sections of the images (Panel D, L, T). Percentage of bacteria expressing Aap on the surface from the total bacteria population of 5–6 microscopic fields, of at least three independent experiments, is shown in panel U. Asterisks indicate significant difference by one-way ANOVA Bonferroni’s post-test (**, P < 0.001; ***, P < 0.0001).

Structural similarities between AatD and Lnt suggest that the latter could complement the 042*aatD* strain. To test our hypothesis, *lnt* (termed Lnt_042_, CBG33518.1) was cloned into the low copy plasmid pBAD30 under the P_*aar*_ promoter for constitutive expression [34]. The 042*aatD* strain was transformed with pLnt_042_ and grown in DMEM-HG and the periplasmic fraction was extracted and analyzed by SDS-PAGE and WB. We observed that like pAatD, pLnt_042_ restored the trafficking of Aap in the 042*aatD* strain as judged by the poor retention of Aap in the periplasm (Fig 8) and localization of Aap at the bacterial surface (Fig 9Q–9U). Approximately 35% of the whole bacteria population complemented with Lnt expressed Aap at the bacterial surface (Fig 9U).

### Aap is an acylated lipoprotein

**Fig 10:**
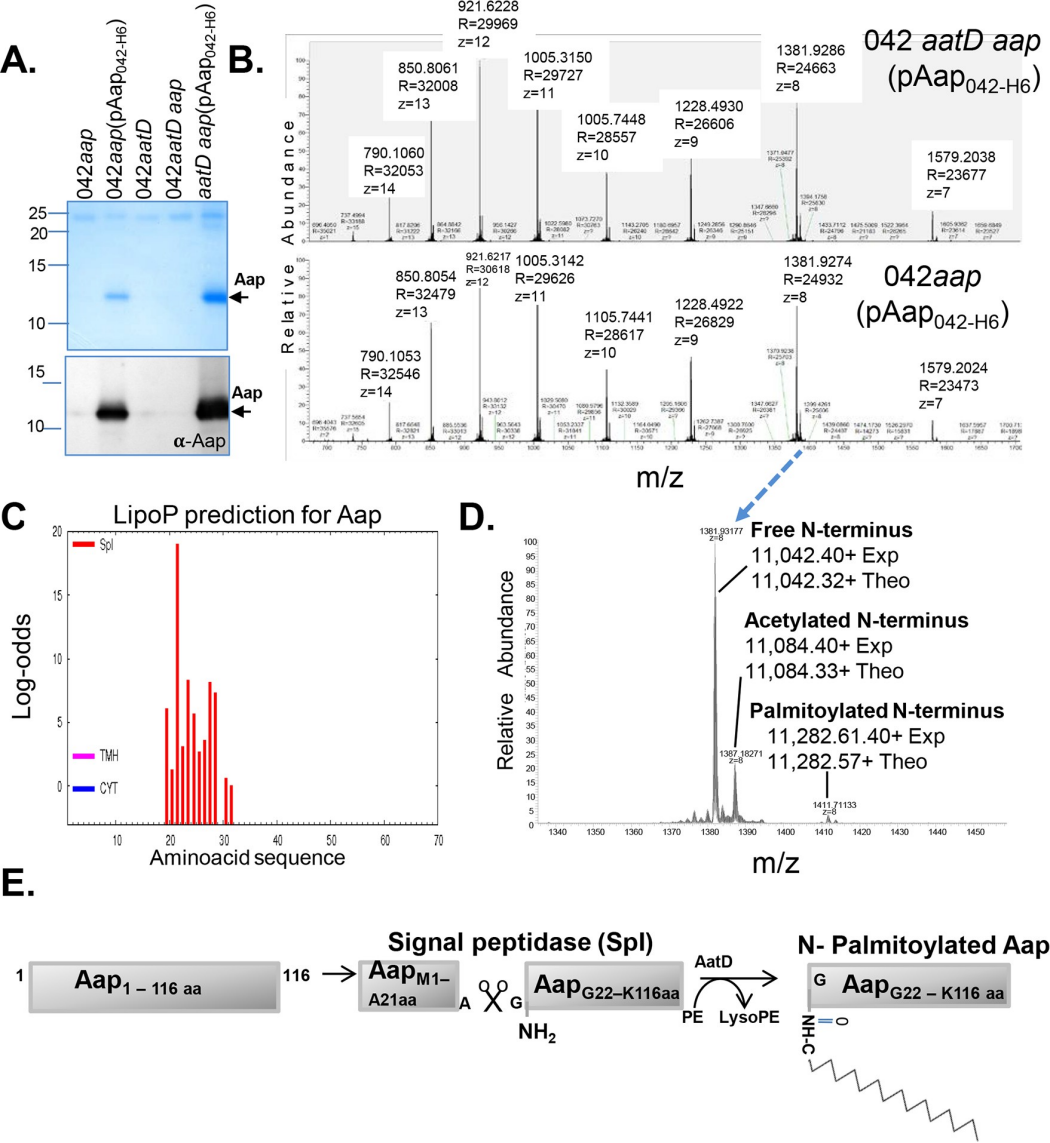
Aap is an acylated lipoprotein. Aap_042-H6_ was purified from 042*aap*(pAap_042-H6_) and 042*aatD aap*(pAap_042-H6_) by nickel affinity columns (Panel A). The samples were analyzed by LC-MS (Panels B and D). A cleavage site for Signal peptidase I was predicted in Aap using the LipoP algorithm (Panel C). Acylation of Aap was confirmed by LC-MS analysis (Panel D). Posttranslational modification of Aap is illustrated in Panel E.

Acylation of Aap could explain the result in changes of Aap membrane association, localization, and trafficking in the cell. We sought to identify the post translational modification of Aap in presence of AatD. Accordingly, we purified Aap_042-H6_ recombinant proteins from cell lysates of 042*aap*(pAap_042-H6_) and 042*aatD aap*(pAap_042-H6_) using nickel affinity columns (Fig 10A). The samples were analyzed by LC-MS and MS/MS (W. M. Keck Biomedical Mass Spectrometry Lab, University of Virginia) (Fig 10B–10D). The intact molecular weight scan for 042*aap*(pAap_042-H6_) and 042*aatD aap*(pAap_042-H6_) is shown in Fig 10B. The major product in both conditions has a monoisotopic molecular weight of 11,042.40^+^ which corresponds to a cleavage between Ala_21_-Gly_22_ and continuing through the 6His-tag. Accordingly, a cleavage site for the Signal peptidase I and lipobox were predicted in Aap using the LipoP prediction algorithm (Fig 10C) [36].

An additional satellite peak corresponds a molecular weight of 11,084.40^+^ which would indicate an acetyl addition. These mass species are identical in both conditions and account for ~99% of each sample. The lower abundance satellites appear to be mostly oxidations and in-source fragmentation (degradation in the instrument high pressure region). One difference was found in the 042*aap*(pAap_042-H6_) sample corresponding to a molecular weight of 11,282,61^+^ (~1% abundance–matching palmitoyl addition) (Fig 10D). No other fatty acid possible additions were found.

Aliquots from both samples were digested with trypsin to produce peptides for confirmation of the N-terminal cleavage and localization of posttranslational modifications (PTMs).S4A Fig shows an N-terminal peptide beginning with Gly_22_ (first 21 amino acids never observed) through Lys38 with a free amino terminus. S4B Fig shows the same peptide with a +42Da mass addition corresponding to an acetylated N-terminus protein. Both of these peptides are observed in both samples. S4C Fig shows a peptide with a +238Da mass addition. This peptide is only observed in the 042*aap*(pAap) sample and corresponds to a protein N-terminal palmitoylation. The posttranslational modification of Aap is illustrated in Fig 10E.

### Genomic rearrangements of *aat* operons and conservation of AatD function

**Fig 11:**
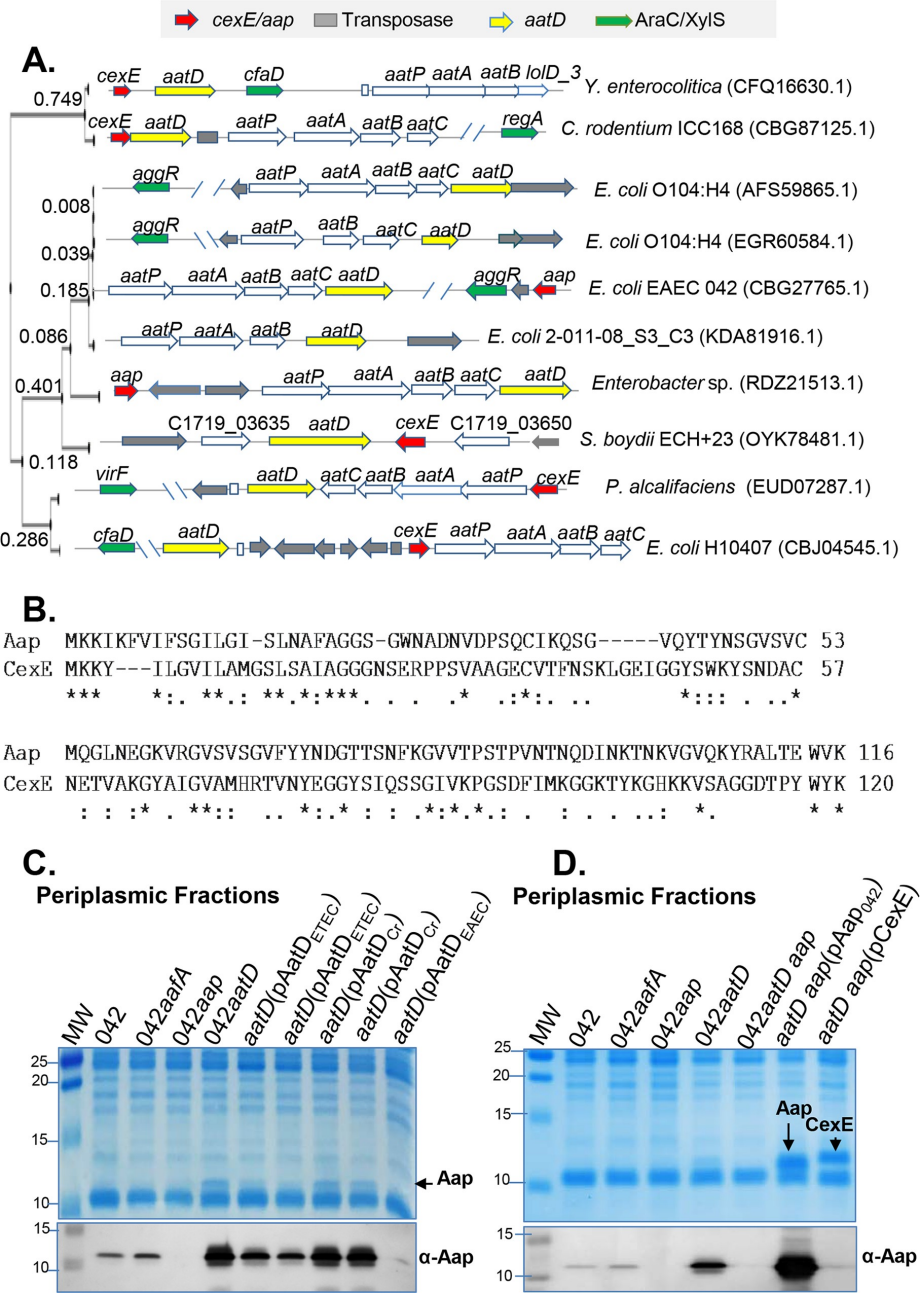
Genomic rearrangements of the *aat* operon and conservation of AatD function. Illustration of the genomic rearrangements of the *aat* operon in the order Enterobacterales (Panel A). The Aap and CexE proteins share 23.7% identity and 61.3% homology in 93 aa overlap (Panel B). Periplasmic fractions of 042*aatD* complemented *in trans* with encoding *aatD* genes of EAEC (*aatD*_*EAEC*_), ETEC (*aatD*_*ETEC*_) and *C*. *rodentium* (*aatD*_*Cr*_) were analyzed by coomassie blue SDS-PAGE and western blot using a polyclonal antibody against Aap (Panel C). Periplasmic fractions of 042*aatD aap* transformed with plasmids encoding the homologous Aap of ETEC (CexE) and EAEC strain 042 (Aap_042_) were analyzed as in Panel D. Of note that despite the 61.3% of similarity between of both Aap and CexE proteins, anti-Aap antibodies did not recognize CexE protein (Panel D, WB with anti-Aap).

Diverse gene organization and high genetic variability (49 to 54% similarity) was observed in the *aat* operon (Fig 11A). Conservation of protein structure would presumably dictate conservation of AatD function across the family. We sought to determine whether AatD homologs from ETEC (termed AatD_*ETEC*,_ CBJ04545.1) and *C*. *rodentium* (termed AatD_*Cr*,_ CBG87125.1) could trans-complement the 042*aatD* strain (Fig 11C). AatD encoding genes were cloned into pBAD30 plasmid under the P_*aar*_ promoter [34]. 042*aatD* was transformed with pBAD30 derivatives (pAatD_*EAEC*,_ pAatD_*Cr*_ and pAatD_*ETEC*_) and grown in DMEM-HG. Periplasmic fractions were obtained and analyzed by SDS-PAGE and WB. We observed that AatD_*ETEC*_ was partially capable of complementing 042*aatD* as judged by the poor retention of Aap in the bacterial periplasm, while AatD_*Cr*_ failed to complement 042*aatD* (Fig 11C).

Likewise, we sought to determine if Aap homolog from ETEC strain H10407 (termed CexE, CBJ04537.1) could be targeted by AatD in EAEC. Accordingly, 042*aatD aap* strain was *trans*-complemented with either a plasmid expressing the ETEC strain H10407 CexE protein (pCexE) or with the plasmid expressing the 042 Aap protein (pAap_042_) (Fig 11B). Strains were grown in DMEM-HG and prepared for periplasmic extraction. A ~12kDa protein band corresponding to Aap in 042*aatD aap*(pAap_042_) and CexE in 042*aatD aap*(pCexE) was observed in the coomassie-stained gel (Fig 11D), suggesting that Aap and CexE are accumulated in the periplasm of 042*aatD aap*. Of note, antibodies raised against Aap_042_ did not cross-react with CexE (Fig 11D).

### Evaluation of 04*aatD* mutant in the streptomycin-treated mouse model

Since Aap homologs are expressed within virulence regulons of several species of enteric pathogens and required for colonization of murine intestinal tissues by *C*. *rodentium in vivo* [37]. We sought to determine if affecting Aap trafficking to the external milieu in 042*aatD* could affect bacterial colonization using the streptomycin-treated mouse model (ST-model) [38]. In this model, 042 persists in the intestine up to 15 days. Although both the ileum and colon are colonized by 042, the colon exhibits greater colonization by the strain [38].

**Fig 12:**
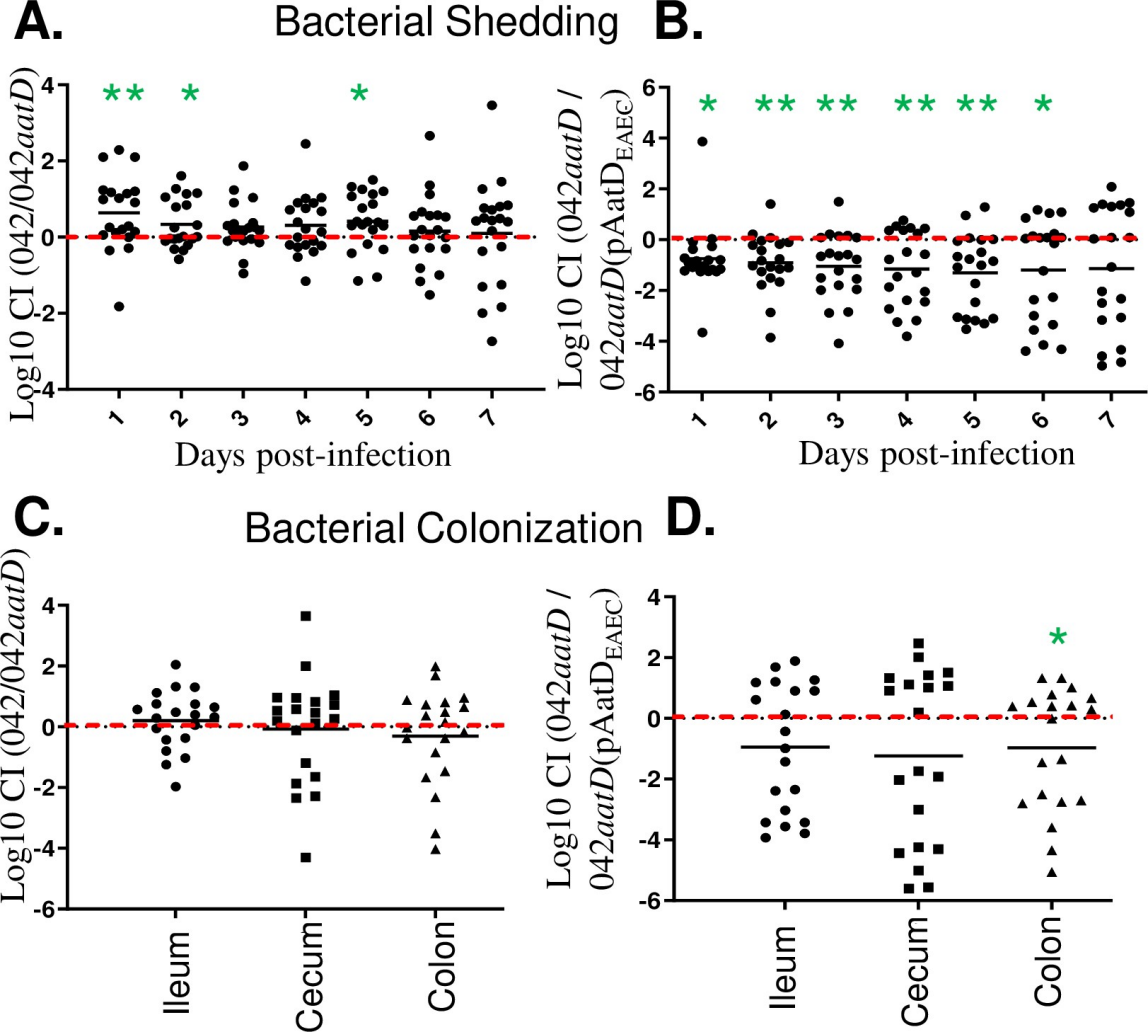
Evaluation of 04*aatD* mutant in the streptomycin-treated mouse model. The streptomycin-treated mouse model was used to evaluate the role of AatD *in vivo*. Groups of 10 C57BL/6 mice were coinfected with 10^7^ cfu/ml of 042 / 042*aatD* (Panels A and C) and 042*aatD* / 042*aatD*(pAatD_EAEC_) (Panels B and D). Bacterial shedding was determined every day for 7 days (Panel A and B). Mice were euthanized on day 7 post-inoculation to determine bacterial colonization of ileum, cecum and colon (Panels C and D). The competitive index (CI) was determined by the ratio of Wt 042 to 042-derivative recovered from mice compared to the ratio of bacteria in the inoculum. Mutants with a CI of < -0.5 or > +0.5 are considered less-colonizer or hyper-colonizer, respectively. Data are representative of two independent experiments. Asterisks indicate significant difference by T- test (*, P < 0.01; **, P < 0.001).

Accordingly, groups of 10 mice were coinfected with 10^7^ cfu/ml of 042 / 042*aatD* and 042*aatD* / 042*aatD*(pAatD_EAEC_) as described in material and methods. Bacterial shedding was determined every day for 7 days (Fig 12A and 12B). Mice were euthanized on day 7 post-inoculation to determine bacterial colonization of the ileum, cecum and colon (Fig 12C and 12D). Statistically significant differences in bacterial shedding were observed at early time points of infection (Fig 12A). Our finding suggests that the presence of AatD makes 042 strain a better colonizer.

## Discussion

The master regulator of virulence AggR and its negative regulator Aar modulate the expression of several virulence factors in EAEC including fimbriae, toxins, T3SS, T6SS, Aap and the AatPABCD operon required for transport of dispersin to the bacterial surface [25]. In this study, we show that the AggR/Aar regulatory system also regulates the metabolism of lipids. Metabolomics analysis revealed that 042*aar*, which has unrepressed expression of AggR, has elevated levels of phosphatidylethanolamine (PE) and lysophosphatidylethanolamine (LysoPE) species when compared to Wt 042 (Fig 1).

PE and lysoPE lipid species are essential components of the bacterial membrane and have an important role in the proper assembly of proteins into the OM [39]. Since these molecules are also substrates and degradation products in the N-acylation of apolipoproteins by action of N-acyltransferases, we hypothesized that perhaps N-acyltransferases are also regulated by the AggR/Aar system. In the search for these enzymes in 042, we took advantage of previous RNA-seq and microarray data derived from 042*aggR* and 042*aar* mutants, which identified a hypothetical N-acyltransferase under AggR/Aar control previously annotated as AatD [8, 24].

AatD is part of the ABC transporter complex designated AatPABCD, required for the transport of Aap. This transporter includes an inner-membrane permease (AatP), an ATP-binding cassette protein (AatC), AatB (unknown function) and the outer membrane TolC-like protein (AatA). Although the components of this channel were partially characterized previously, the function of AatD in the *aat* operon remained elusive [25]. In silico analysis revealed structural similarities between AatD and N-acyltransferase Lnt [40]. It is well documented that depletion of the Lnt causes mislocalization of outer membrane lipoproteins in *E*. *coli* [22, 32], while deletion of *aatD* caused retention of Aap in the bacterial periplasm [25]. We found that Aap not only is retained in the periplasm of 042*aatD*, but is present in a higher amount in whole cell fractions of 042*aatD* than Wt 042 (Fig 4A). We therefore sought to determine more extensively how *aatD* affects trafficking of Aap in the 042 strain. We found that Aap is exported to the bacterial surface in approximately 40–50% of Wt 042 bacteria and its transport depends on the presence of AatD (Figs 5 and 9). We also found Aap localized mostly at the bacterial poles.

Polar trafficking findings were confirmed by using the Aap-mCherry reporter protein. In these studies, Aap-mCherry was accumulated in the periplasmic poles of 042*aatD* and colocalized with the bacterial membrane in 042*aap*, which bears a functional AatD (Fig 6H–6L and S3 Fig). Regarding the percentage of bacteria expressing Aap at the bacterial surface, a study using the homologous Aap of *C*. *rodentium* CexE found similar results, not all bacteria export CexE to the bacterial surface [37].

Since we observed relative higher expression of Aap in the 042*aatD* than Wt 042 in whole cell extract, it is tempting to hypothesize that perhaps the relative abundance of exported lipoproteins in bacteria is a tightly regulated process, and that beside re-directing lipoproteins to the outer membrane, acylation could be also a signal that target lipoprotein degradation [41]. In this scenario, Aap in the 042*aatD* may have escaped this protein quality control process. Additional experiments need to be carried out to test this assumption.

We found that the export of Aap to the surface of the 042*aatD* mutant could be also restored by Lnt and the homologous AatD of ETEC (Figs 8 and 11). Moreover, mutation of E207 residue in the putative catalytic triad of AatD caused accumulation of Aap in the periplasm and affected its surface display (Figs 7 and 9). Lastly, we demonstrated that Aap is a lipoprotein acylated by AatD at the N-terminus (Fig 10 and S4 Fig). Taken together, these data demonstrate that AatD is an N-acyltransferase required for the efficient transport of Aap in EAEC.

**Fig 13:**
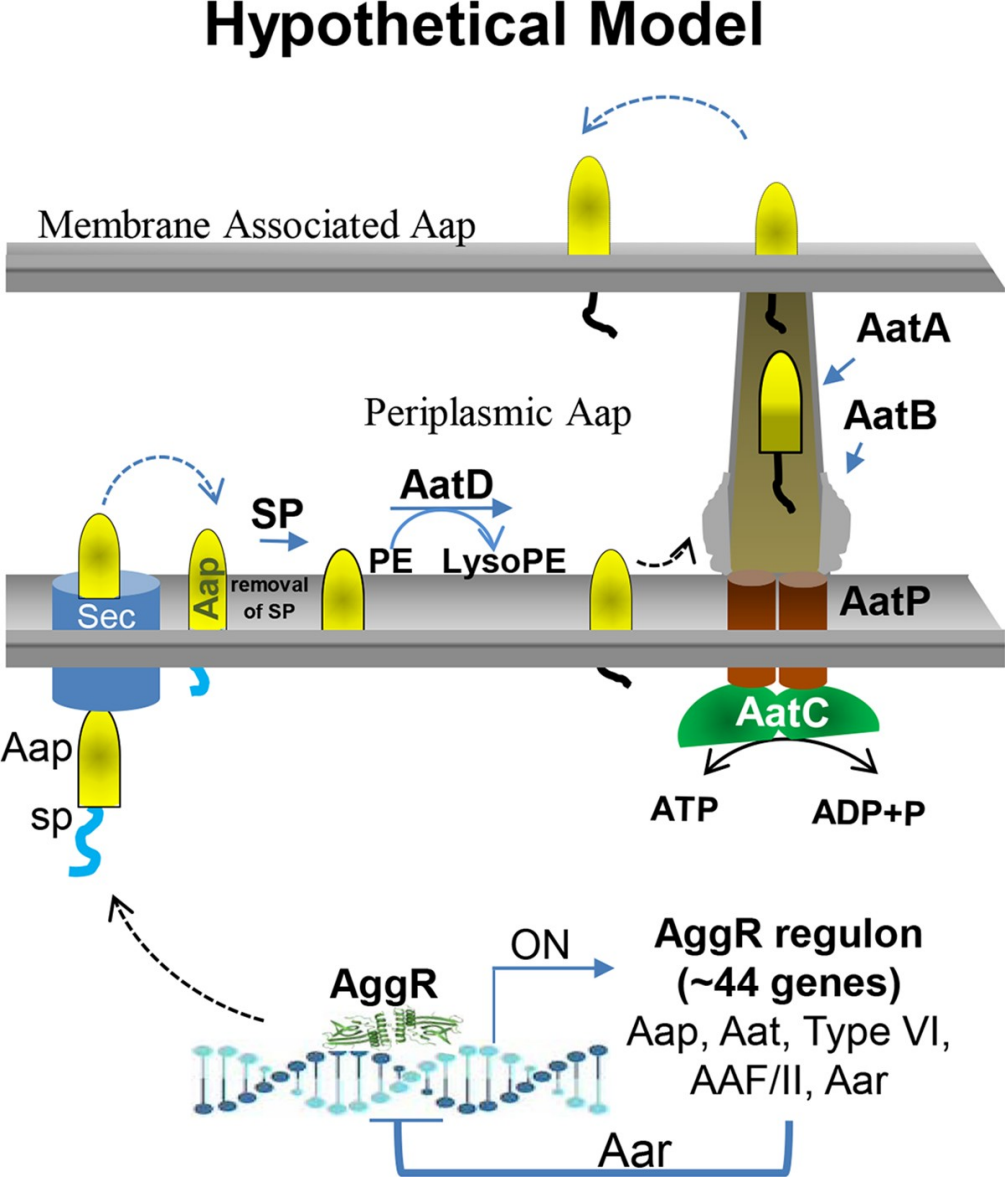
Model of Acylation of Aap in EAEC. The hypothetical model was constructed based on previously published Aat and Lnt translocation models. The model depicts the synthesis of AatPABCD operon in the bacterium cytoplasm following the activation of AggR. Aat proteins are translocated into the periplasm by the Sec-apparatus, where AatA, AatB and AatP assemble into the Aat channel, while AatC provides the energy to activate the Aat translocon. Aap is acylated at the inner membrane by AatD. Presumably, proper acylation of Aap ensure its translocation through the Aat channel toward the bacterial surface.

We provided a hypothetical model of Aap trafficking in EAEC based on our experimental data and published translocation models of Aat and Lnt (Fig 13) [14, 25]. In this model, following activation of AraC/XylS AggR regulator, Aat proteins are synthesized and translocated into the periplasmic space by the Sec-apparatus for the ensemble of the Aat channel. Acylation of Aap by AatD may take place in the periplasmic side of the inner membrane. Presumably, proper acylation of Aap ensure its translocation through the Aat channel toward the outer membrane.

Several questions remain to be answered on the relevance of the acylation of Aap, e.g.; whether acylation of Aap is necessary for recognition of Aap by the AatABC translocator or for its interaction with the TolC-homolog AatA at the OM. Confocal microscopy analysis using the Aap_59-cherry_ reporter system shows that the absence of the AatC ATPase causes diffusion of Aap_59-cherry_ protein in the periplasm with few focal point of Aap-mCherry at the poles (Fig 6 and S3 Fig), perhaps as the result of the untargeted form of Aap in a non-functional Aat translocator. Additional experiments are needed to elucidate the relationship between the post-translational modification of Aap in its targeting to the Aat channel and whether the Aat apparatus is assembled at the bacterial poles.

Lastly, we sought to define the relevance of AatD in bacterial pathogenesis. Given that there is no animal model that recapitulates all aspects of disease for EAEC, we used the streptomycin–treated mouse model that has proven to be an effective method to study colonization traits, metabolic fitness, and *in vivo* gene expression [38, 42–48]. We found that AatD was required for efficient colonization of the mouse gastrointestinal tract in competition infections in this model.

To our knowledge this is the first study characterizing a member of the AraC-family that modules virulence at two levels: 1) by controlling gene transcription levels of virulence factors and 2) by controlling the post-translational modification of these factors through regulation of its own N-acyltransferase; an important trait for the full virulence of EAEC and perhaps for the virulence of many other pathogens.

## Materials and methods

### Bacterial strain and growth conditions

Bacterial strains used in this study are shown in S2 Table. EAEC 042 derivatives were routinely propagated in Luria Broth (LB) and Dulbecco’s modified Eagle’s medium with 0.4% glucose (DMEM high glucose) (Gibco, Grand Island, NY) as previously described [8, 34]. For periplasmic isolation, the bacteria were propagated on DMEM plates with 0.2% glucose (DMEM-high glucose plates) at 37°C [49].

### Generation of 042 derivatives

Mutagenesis of 042 derivatives was accomplished by using lambda red technology [50]. The locus for *aap* (CBG27807.1), *aatC* (CBG27764) and *aatD* (CBG27765.1) in the pAA2 plasmid (GenBank FN554767.1) were replaced by the kanamycin (km) resistance marker. Primers used for the lambda-red procedure are indicated in S3 Table. For complementation *in trans*, *aatD*_EAEC_, *aatD*_ETEC_, *aatD*_Cr_, *aap*_042_, *cexE* and *aap*_Cr_ genes were amplified by PCR and cloned into pBAD30 derivative plasmid under P_*aar*_ promoter as previously reported [34].

### Site Directed Mutagenesis of the AatD catalytic triad

Amino acids Glu(E)_207_, Lys(K)_268_ and Cys(C)_316_ were replaced by Alanine (A) by using the QuikChange II XL Site-Directed Mutagenesis Kit (Agilent Technologies, CA USA). As a template for the mutagenesis reaction, 10 ηg of pAatD plasmid was mixed with 125 ηg of the corresponding primers (207EAsen, 207EArev, 268KAsen, 268KArev, 316CAsen and 316CArev) (S3 Table) and 2.5 U/μl of Pfu Turbo DNA polymerase. Samples were treated for 1 cycle/3 minutes at 95°C followed by 18 cycles of 95°C/30 seconds, 55°C/1 minute, 68°C/ 8 minutes. Samples were digested with *Dpn*I for 2 h at 37°C and transformed into XL10-Gold Ultracompetent Cells (Agilent Technologies, CA USA). Mutation of Lys(K)_268_ was toxic for the bacteria. The other resulting plasmids were designated pAatD_E207A_ and pAatD_C316A_ respectively. All constructs were verified by nucleotide sequencing.

### Metabolomic analysis

EAEC 042 and 042*aar* derivatives were propagated in 14 ml of DMEM high glucose for 6 h as previously described [8, 34]. Bacterial pellets were washed in 1 ml of sterile PBS and analyzed by Creative Proteomics Inc. Briefly, the samples were lysed by incubation of 5 minutes at -80°C and 5 minutes at 37°C. The incubation cycles were repeated five times. Samples were centrifuged at 15,000 rpm for 10 minutes. 200 μl of supernatant were mixed with 600 μl of acetonitrile, vortexed for 30 seconds and centrifuged for 15000 rpm for 10 minutes. 100 μl of supernatant were used for LC/MS metabolomics analysis. The LC/MS analysis platform (Waters UPLC Class 1 TOF/Q-TOF Mass Spectrometer), column (Waters ACQUITY UPLC HSS C18 column (10 cm × 2.1 mm × 1.7 μm), parameters (Gas temperature: 450°C; Gas flow: 13 L/min; Nebulizer: 35 psi; Sheath; Gas temperature: 500°C; Sheath Gas Flow: 15 L/min; capillary voltage: 3000 V (positive mode) and 2000 V (negative mode); nozzle voltage: 1000 V; fragmentor: 175 V) was previously standardized by Creative Proteomics Inc. The raw data were acquired and aligned by using the Makerlynx software (version 4.1) based on the m/z value and the retention time of the ion signals. Metabolites were identified by Mass Bank database. Means of peak intensity of individual metabolites were normalized to wild type 042 from two independent experiments run in duplicate, and analyzed by unpaired T-test statistical analysis. A *p* value of less than 0.05 was considered statistically significant.

### Periplasmic isolation

Periplasmic fractions were isolated from DMEM plates with 0.2% glucose [49]. Bacterial pellets were washed with 1 ml of PBS and resuspended in 1 ml of periplasmic solution (20% sucrose, 30 mM Tris-HCl pH 8.0, 1 mM EDTA pH 8.0). Bacterial preps were incubated for 20 min at room temperature, pelleted and resuspended in 200 μl of ice-cold 5mM MgSO_4_. Bacterial preparations were incubated for 20 min at 4°C and centrifuged at 14,000 rpm for 5 min. Supernatants were isolated and analyzed by SDS-PAGE and WB.

### Preparation of membrane fractions

EAEC 042 derivatives were propagated in 14 ml DMEM high glucose for 6 h as previously described [8, 34]. Bacterial pellets were obtained and washed in 1 ml of sterile PBS. Bacterial pellets were resuspended in lysis buffer (20 mM Tris, pH 8.0, 500 mM NaCl, and 1x protease inhibitor) and lysed by sonication. Cellular debris was removed by centrifugation at 18,000 g for 15 min at 4°C. The membrane fraction was isolated by ultracentrifugation at 125,000 g for 1 h at 4 ^o^C. The membranes were analyzed by SDS-PAGE and WB.

### Analysis of Aap-mCherry compartmentalization by confocal microscopy

Compartmentalization of Aap was determined by confocal microscopy. For these studies, pAap_59-cherry_ plasmid was generated by fusing the encoding region of 59 N-terminal amino acids of Aap to *mcherry* gene. The fusion Aap_59-cherry_ was constitutively expressed under *P*_*aar*_ promoter [34]. 042 derivatives (042*aap*, 042*aatD aap* and 042*aatD aatC*) were transformed with pAap_59-cherry_ and analyzed by confocal microscopy. As positive control of trafficking, we generated pLpp_23-cherry_ plasmid encoding the first 23 amino acids of lipoprotein Lpp fused to *mCherry* gene [35]. EAEC 042 derivatives were transformed with pAap_59-cherry_ or pLpp_23-cherry._ The bacteria were propagated on DMEM plates with 0.2% glucose (DMEM-high glucose) at 37 ^o^C for 24 h [49]. A small bacterial pellet was resuspended in 100 μl of sterile PBS with 1 μM of CellBrite dye (Biotium, Fremont, CA) and 1μg/ml of Hoechst 33342 dye (Biotium, Fremont, CA). Samples were incubated for 1 h at 4 ^o^C, washed in 1 ml of PBS, and prepared for confocal analysis. Confocal microscopy was performed with Zeiss LSM 710 Multiphoton Confocal system (Zeiss, Germany) at the Advanced Microscopy Facility of UVA. The samples were analyzed for GFP signal (488-ηm), UV laser for DNA (363-ηm) and the red channel (543-ηm). The resulting images were overlaid by using ZENlite 2012 software (Zeiss, Germany). Colocalization of red and green signal produced a yellow color.

### Detection of Aap at the bacterial surface by confocal microscopy

For surface detection of Aap, we performed indirect-immunofluorescence as follows: 042 derivatives were grown on DMEM-HG agar plates as described above. Cells were harvested and resuspended in 1ml of PBS (~ OD_600_ = 1.0/ml), washed twice with 1ml of PBS and resuspended in 1 ml of blocking solution (1% BSA in PBS). Cells were incubated at RT for 1h with rocking. Subsequently, cells were pelleted and incubated with a rabbit polyclonal anti-Aap antibody in 300 μl of blocking solution (1:100 dilution) for 1h with rocking. Cells were washed twice with 1ml of PBS and incubated with Alexa-488 or Alexa-594-conjugated anti-rabbit IgG (Invitrogen), with or without 1μM of CellBrite dye (Biotium, Fremont, CA) and 1μg/ml of Hoechst 33342 dye (Biotium) in 300 μl of blocking solution (1:500 dilution) for 1h with rocking. Then, cells were washed twice with 1 ml of PBS and resuspended in 100 μl of PBS. 10 μl of the bacterial suspension was spread in glass slides with coverslips and analyzed by confocal microscopy using the 64X immersion oil objective. Images were acquired using the ZENlite 2012 software (Zeiss, Germany). The percentage of bacteria expressing Aap on the surface from the total bacteria population of 5–6 microscopic fields, of at least three independent experiments, was obtained by using ImageJ particle enumeration algorithm [51]. Briefly, the number of bacteria expressing Aap on the surface in each microscopic field was first obtained by enumerating Alexa 488-conjugated secondary antibody-stained bacteria or Alexa 594-conjugated secondary antibody-stained bacteria. Then, this number was correlated with the total number of bacteria in the same microscopic field, obtained by enumerating Hoechst 33342-stained bacteria or CellBrite-stained bacteria.

### Aap purification

pAap_042-H6_ plasmid was engineered to encode Aap fused to His_6_-Tag. The plasmid was transformed into 042*aap* and 042*aatD aap*. The strains were grown in DMEM high glucose medium. Bacterial pellets were sonicated and supernatant used for the protein purification. For 500 ml of medium, we prepared 10 ml of nickel beads. The beads were washed and equilibrated in His-binding Buffer at room temperature (50 mM Tris-Cl pH 8.0, 5 mM Imidazole, 100 mM NaCl, 0.1 mM EDTA). Nickel beads were combined with bacterial lysates and incubated for 1 h at room temperature. Beads were packed in a 50 ml column and washed with 200 ml of Ni-column His-washing buffer (50 mM Tris-Cl pH 8.0, 15 mM Imidazole, 300 mM NaCl, 0.1 mM EDTA). Aap_H6_ was eluted with 10 ml of His-elution buffer (50 mM Tris-Cl pH 8.0, 300 mM Imidazole, 50 mM NaCl, 0.1 mM EDTA). 1ml fractions were saved and analyzed by SDS-PAGE and WB. The protein was dialyzed in PBS and stored a -20°C.

### Detection and quantification of Aap protein

For detection of Aap_042_, strains were grown in 14 ml of DMEM high glucose to reach an OD_600_ of 0.8. Bacteria were pelleted, and periplasmic fractions were isolated and analyzed by SDS-PAGE and Western blot analysis. Protein samples were separated in 20% acrylamide gels and transferred to Immobilon-P membranes (BioRad, Hercules CA, USA) by using standard protocols. The membranes were incubated overnight with anti-Aap antibodies. As controls, anti-AafA, anti-MBP (Invitrogen) and anti-DnaK (Abcam Inc, Cambridge MA) antibodies were used in this study. The next day, the membranes were washed thrice in PBS-0.1% tween, and incubated for 1h with a horseradish peroxidase-conjugated goat anti-rabbit IgG antibody. Membranes were developed using TMB Membrane peroxidase substrate (KPL, Gaithersburg, MD, USA) following the manufacture’s specifications.

For the ELISA analysis, the samples were generated from bacteria cultured on 14 ml of DMEM high glucose. Bacterial pellets were treated with sucrose solution as indicated above to isolate the periplasmic fraction. Whole bacterial preps were isolated and lysed by sonication on ice. Bacterial preparations were centrifuged and proteins cleared lysates were quantified by Enzyme-linked Immunosorbent Assay (ELISA). ELISA plates were covered with 1 μg of whole or periplasmic preps. The plates were washed thrice in PBS-0.1% tween. Plates were incubated for 2 h at 37°C with anti-dispersin and then washed as indicated above to remove any unbound antibody molecules. Plates were incubated with an anti-rabbit secondary antibody. Following the incubation period and additional washing, plates were developed and the signal quantified.

### Mass spectrometry detection of Aap post-translational modification

Aap_042-H6_ was purified in nickel columns from 042*aap* and 042*aatD aap* strains. The proteins were analyzed by LC-MS and MS/MS. For intact molecular weight measurements, the LC-MS system consisted of a Thermo Electron Q Exactive HF mass spectrometer system with an Easy Spray ion source connected to a Thermo 75 μm x 15 cm C18 Easy Spray column using an elution gradient of 0–80% B over 30 minutes (A– 0.1 M formic acid in water, B– 80% acetonitrile / 20% Buffer A). The instrument parameters were resolution 60K, 3 microscans, target 3E6, max IT 100ms). Spectral deconvolution and mass assignment were made using BioPharma Finder 3.1. For Peptide identifications (MS/MS), the same instrument, column and gradient was used with a Top10 HCD method. The instrument parameters were changed to the following–MS (60K resolution, 1 microscan, 3E6 target, max IT 45ms), MS/MS (15K resolution, 1 microscan, 1E5 target, max IT 100ms, 1.3Da isolation, NCE 28, dynamic exclusion). The data was analyzed using the Sequest search algorithm contained with Proteome Discoverer 2.2 using a parent window of 10ppm, fragment window of 0.02Da, variable modifications (oxidized M, phospho STY, and palmitoylation / myristoylation / acetylation KSTC (peptide NT)), semi tryptic. The search results were initially filtered by loading the data into Scaffold 4.10.0 and using the following cutoffs–xcorr +1>1.8, +2>2.0, +3>2.3, +4>3.0, delta CN 0, peptide prophet 60%. Any spectra that indicated possible NT/CT processing and/or PTMs were manually verified.

### Bioinformatics and statistical analysis

Hypothetical proteins were analyzed by using https://mafft.cbrc.jp/alignment/server/ [28] and http://prodata.swmed.edu/promals3d/promals3d.php [26, 27]. The structure of AatD was predicted by phyre2 algorithm (http://www.sbg.bio.ic.ac.uk/phyre2/html/page.cgi?id=index)) [30]. A signal peptidase site was identified in Aap protein by LipoP prediction software (http://www.cbs.dtu.dk/services/LipoP/).

### Streptomycin-treated mouse model

Groups of 10 male BALB/c mice, 4 weeks old (Jackson Laboratories) were provided with drinking water ad libitum containing 5 g/liter streptomycin for 48 h prior to bacterial inoculation and for the duration of the experiment [38]. 042 derivatives were grown overnight in LB broth, diluted 1:100 in DMEM-high glucose and incubated for 3h at 37°C. Bacterial cultures were adjusted to 10^7^ cfu/ml. the mice were administered with 200 μl of 8% sodium bicarbonate to neutralize the stomach acid prior to bacteria inoculation. Separated cages of mice were orogastrically infected with 100 μl of a 1:1 mixture of 042 / 042*aatD* or 042*aatD* / 042*aatD*(pAatD) inoculum. Fecal pellets were collected aseptically from each mouse daily. The number of viable bacteria per gram of feces was determined by plating serial dilutions of the samples onto media containing appropriate antibiotics. For bacterial colonization, mice were euthanized on day 7 post-inoculation. Cecum, proximal and distal colon compartments were excised and bacterial burden was quantified as described above. The statistical significance of the log_10_ of the competitive ratios was calculated by one sample T-test. Results were considered significant at P < 0.05.

### Ethics statement

Animal experiments were performed in accordance with the Guide for the Care and Use of Laboratory Animals of the National Institutes of Health and with the permission of the American Association for the Assessment and Accreditation of Laboratory Animal Care. The protocol was reviewed and approved by the Institutional Animal Care and Use Committee of the University of Virginia (Protocol No. 3999).

### Statistical analysis

Statistical analysis of data was performed using the GraphPad Prism-6 (GraphPad Software, Inc., CA, USA). Metabolomics data was analyzed with unpaired T-student test. Relative abundance of Aap data determined by ELISA was analyzed by One-way ANOVA with multiple comparison and Bonferroni’s post-test. Competitive colonization data was analyzed with unpaired T-student test. In all analysis, P < 0.05 was considered significant.

## Supporting information

S1 Fig Genetic similarities between AatD/Lnt family. AatD and Lnt homologs were compared with Promals3d algorithm (Panel A to E). The secondary structure of AatD/Lnt family was conserved in the Glu(E)-Lys(K)-Cys(C) catalytic triad region (~200–400 aa) (catalytic triad aminoacids are highlighted in blue). PROMALS3D algorithm strongly predicted the presence of highly conserved αββα structural domains in the catalytic triad region. Aminoacids highlighted in yellow are conserved in AatD from 042 strain.
(PPTX)

S2 Fig Evaluation of Aap in Aat mutants. Accumulation of Aap in the periplasmic space of strains containing individual mutations in *aatA*, *aatC*, *and aatD* was evaluated on 20% SDS-PAGE gels (Panel A) and by Western blot with an anti-Aap antibody (Panel B).
(PPTX)

S3 Fig Aap-mCherry accumulates at the poles in the periplasm of 042*aatD*. 042 derivatives (042*aap*, 042*aatD aap* and 042*aatD aatC*) were transformed with pAap_59-cherry_ and grown statically in DMEM overnight at 37°C. Bacterial cells were harvested, washed with PBS and incubated with CellBrite stain (green, for membrane staining) and Hoechst 33342 stain (blue, for DNA staining) for 1 h. Bacterial cells were analyzed using a LSM-710 laser-scanning confocal microscope (Zeiss, Germany). Representative confocal images taken with the 64X oil objective are shown (Panel A-T). Of note, close-ups of these figures are shown in Fig 6 of the manuscript.
(PPTX)

S4 Fig Mass spectrometry detection of Aap post-translational modification. Samples were digested with trypsin and post-translational modifications were analyzed. The N-terminal peptide beginning with Gly22 (first 21 amino acids never observed) through Lys38 with a free amino terminus (Panel A). The same peptide with a +42Da mass addition corresponding to an acetylated protein N-terminus (Panel B). Supplementary Panel C shows the peptide with a +238Da mass addition. This peptide is only observed in the 042*aap*(pAap) sample and corresponds to a protein N-terminal palmitoylation.
(PPTX)

S1 Table Metabolomic raw data. Untargeted metabolomic analysis was performed in 042 and 042*aar* strains by Liquid chromatography / mass spectrometry (LC/MS). The raw data were acquired and aligned by using the Makerlynx software (version 4.1) based on the m/z value and the retention time of the ion signals. Metabolites were identified in Mass Bank database. Raw data from experiments 1and 2 are listed on Tables 1A and 1B respectively.
(DOCX)

S2 Table Strains used in this study. (DOCX)

S3 Table Primers used in this study. (DOCX)
