# Supplementary figures and images for: Characterization of a novel AraC/XylS-regulated family of N-acyltransferases in pathogens of the order Enterobacterales
Source: PLoS Pathog. 2020 Aug 26;16(8):e1008776. doi: 10.1371/journal.ppat.1008776 (PMC7478709; doi:10.1371/journal.ppat.1008776)

## Slide 1
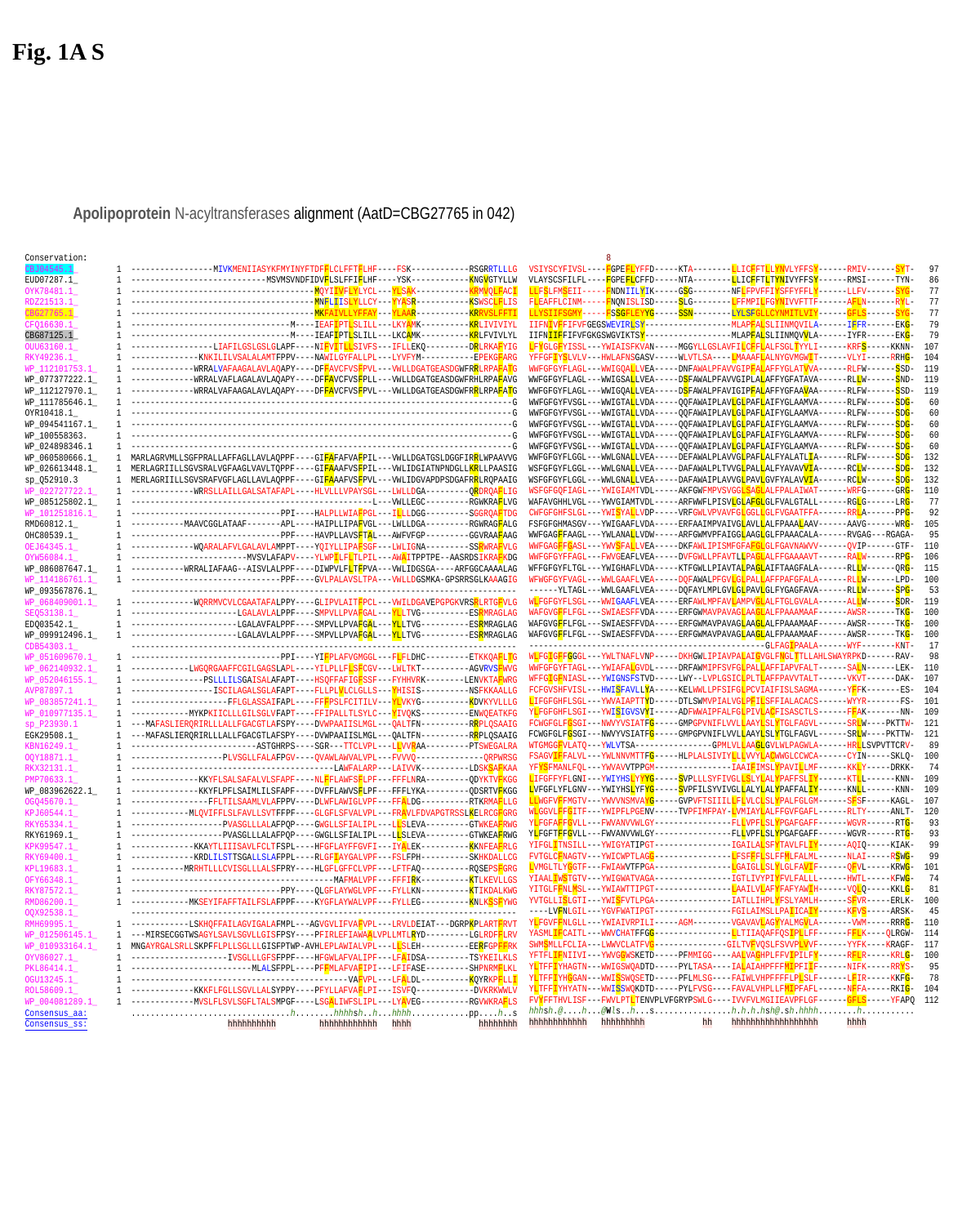

Fig. 1A S

## Slide 2
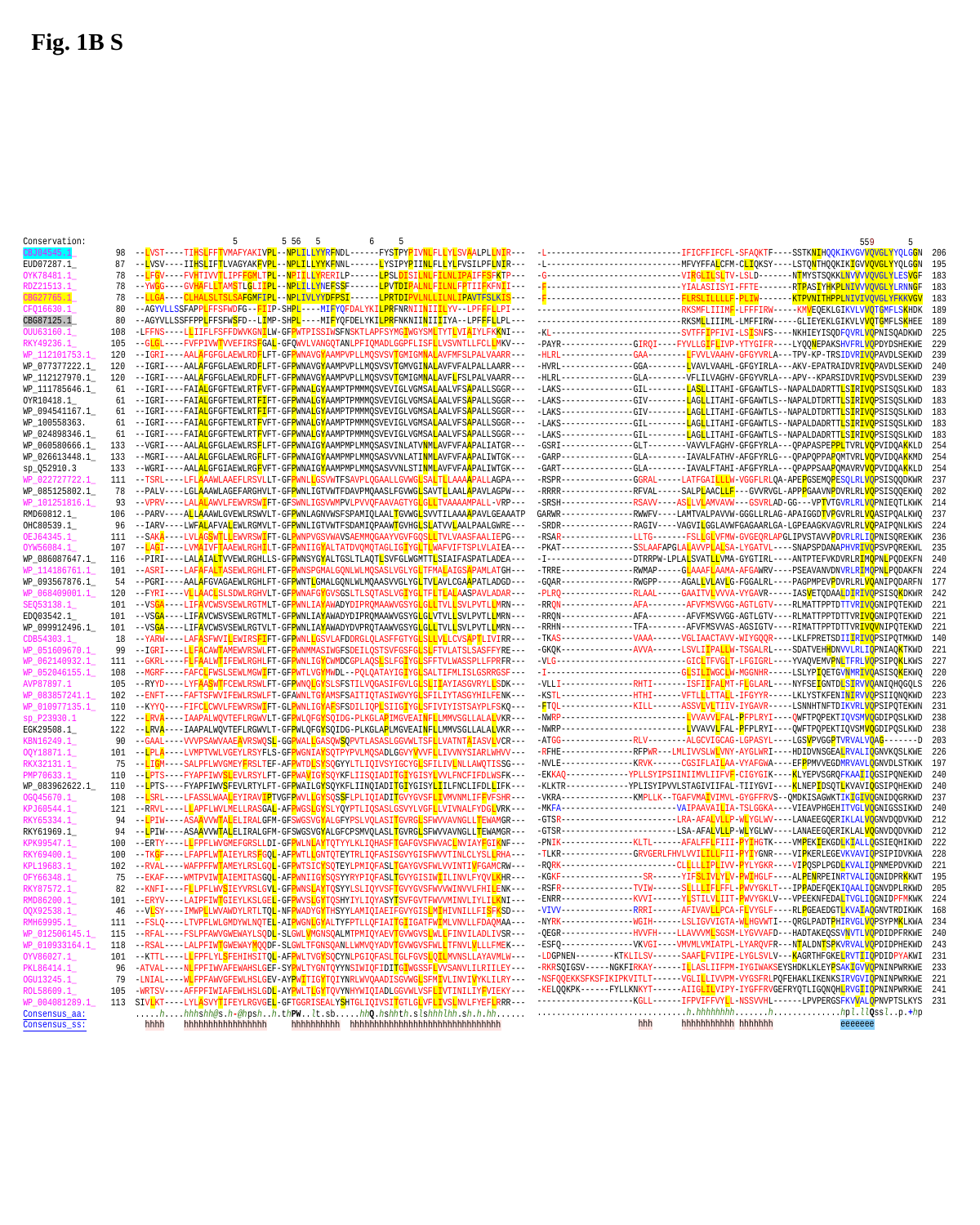

Fig. 1B S

## Slide 3
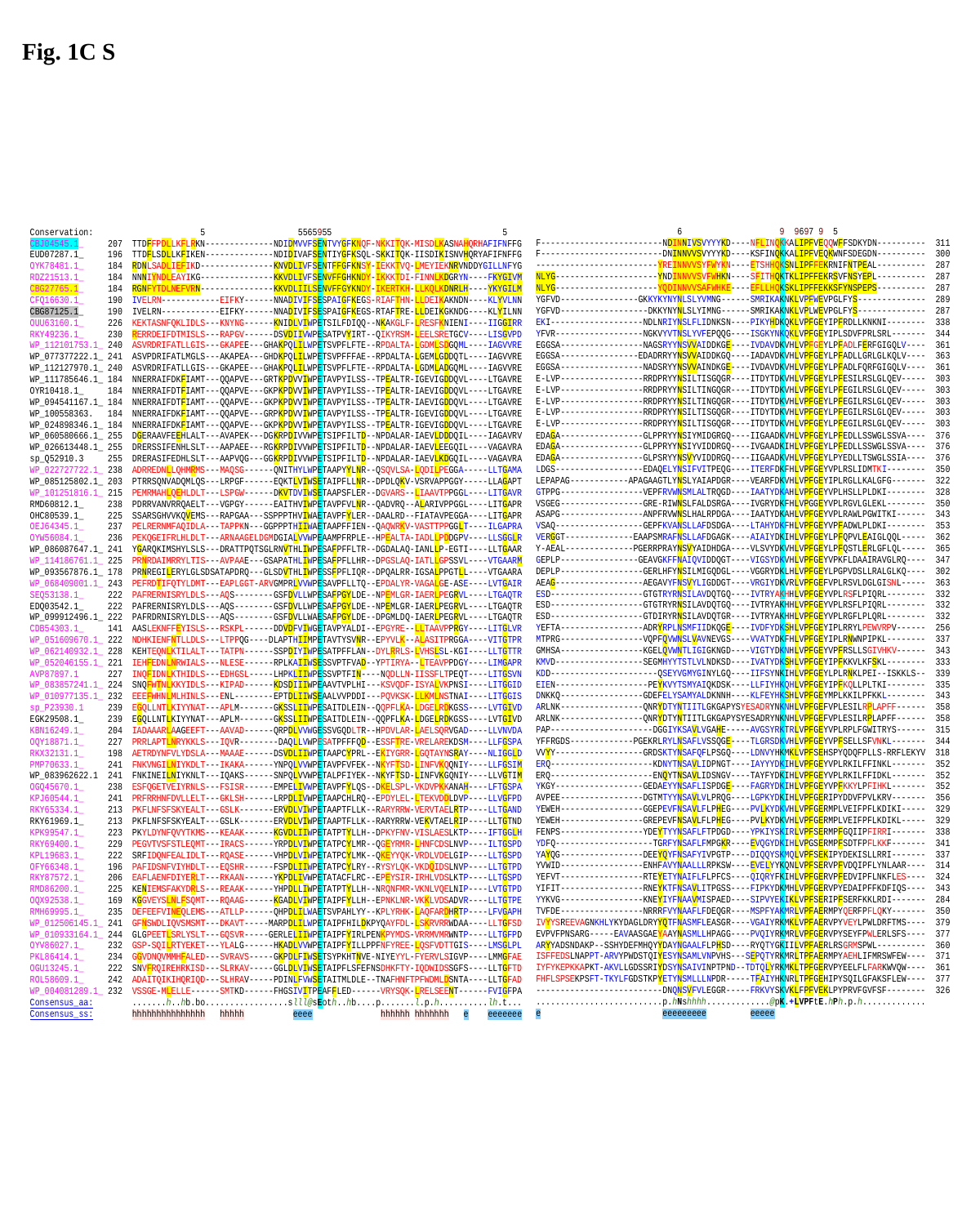

Fig. 1C S

## Slide 4
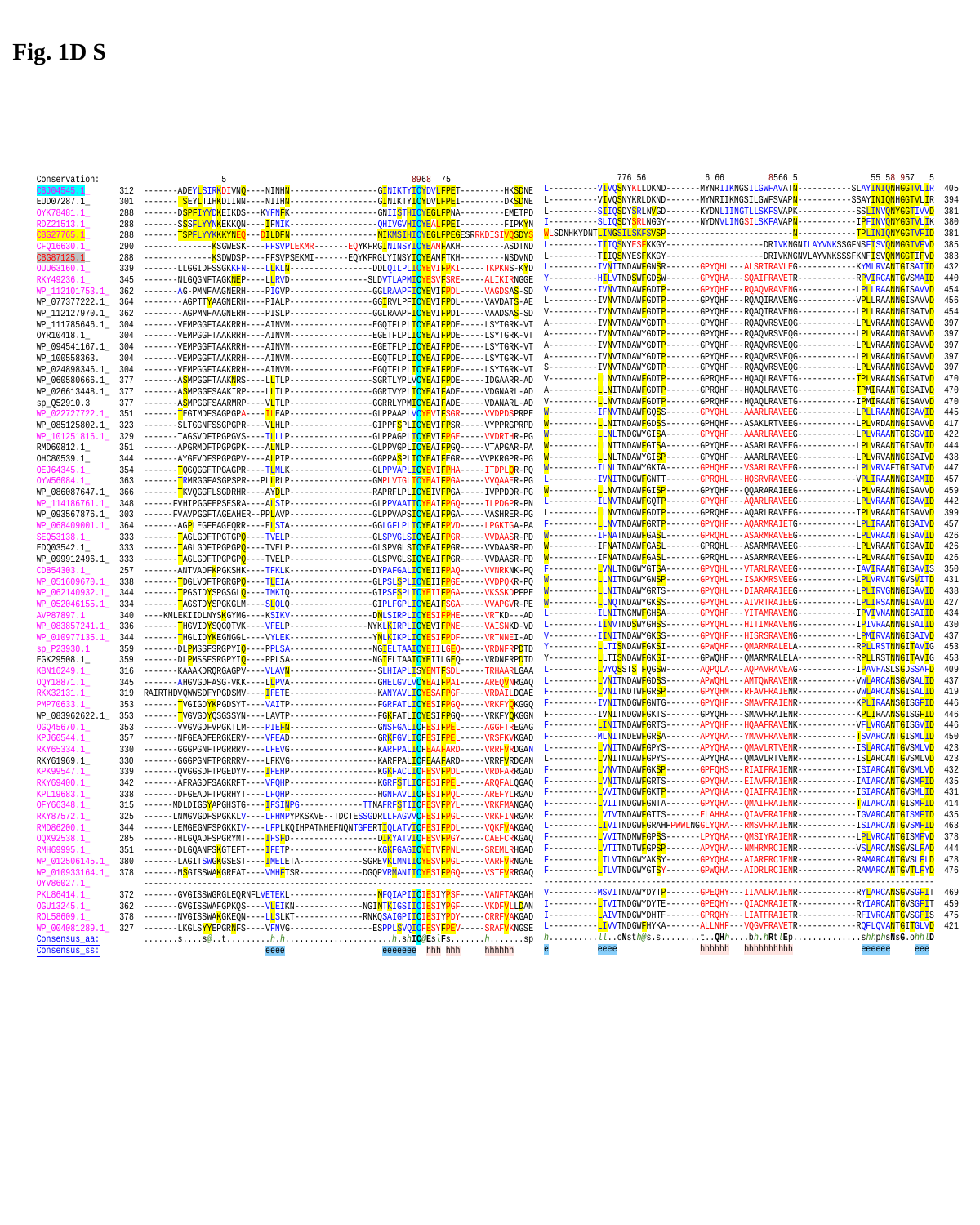

Fig. 1D S

## Slide 5
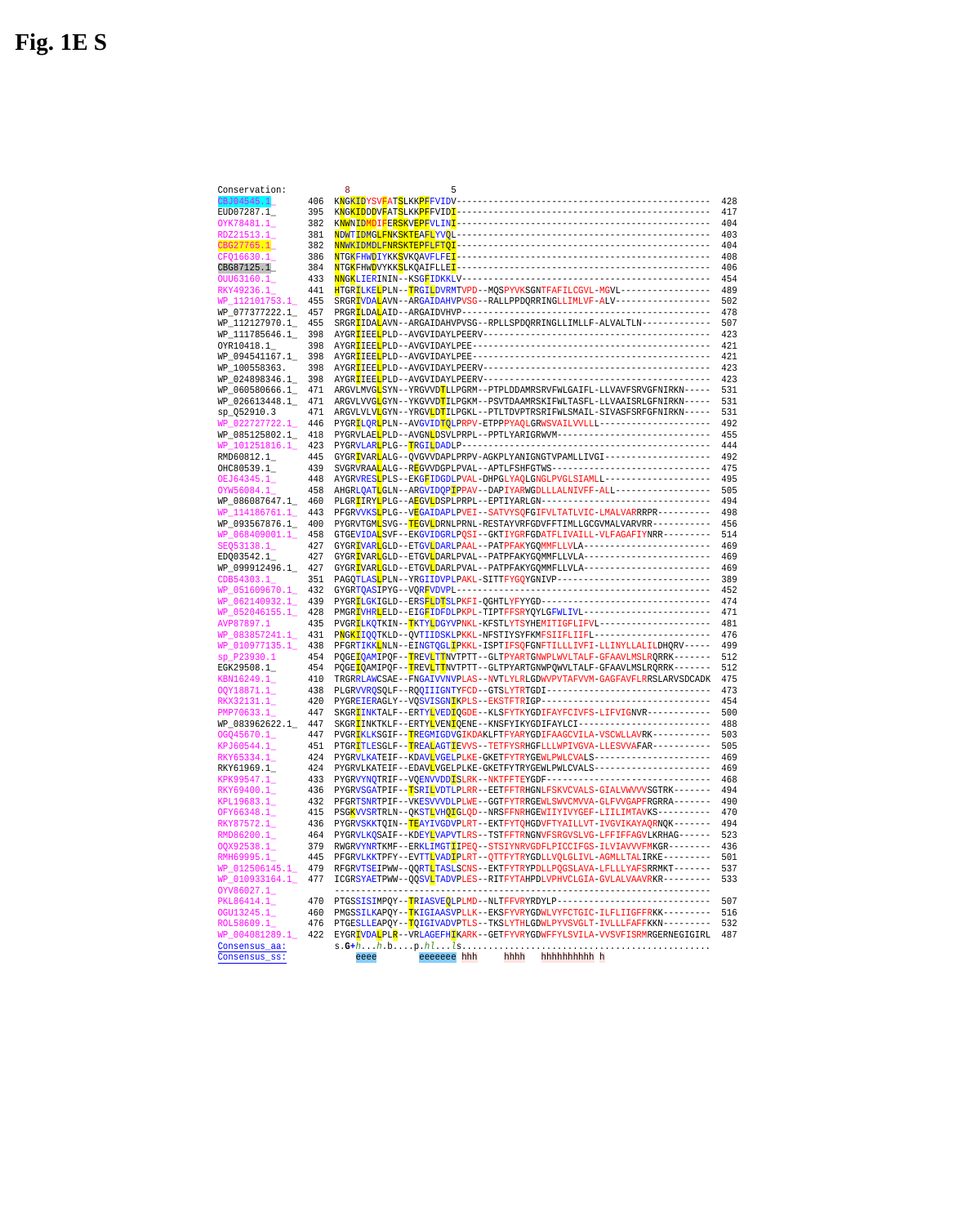

Fig. 1E S

Supplement: S1 Fig — AatD and Lnt homologs were compared with Promals3d algorithm (Panel A to E). The secondary structure of AatD/Lnt family was conserved in the Glu(E)-Lys(K)-Cys(C) catalytic triad region (~200–400 aa) (catalytic triad aminoacids are highlighted in blue). PROMALS3D algorithm strongly predicted the presence of highly conserved αββα structural domains in the catalytic triad region. Aminoacids highlighted in yellow are conserved in AatD from 042 strain. (PPTX) [file ppat.1008776.s001.pptx]

## Slide 1
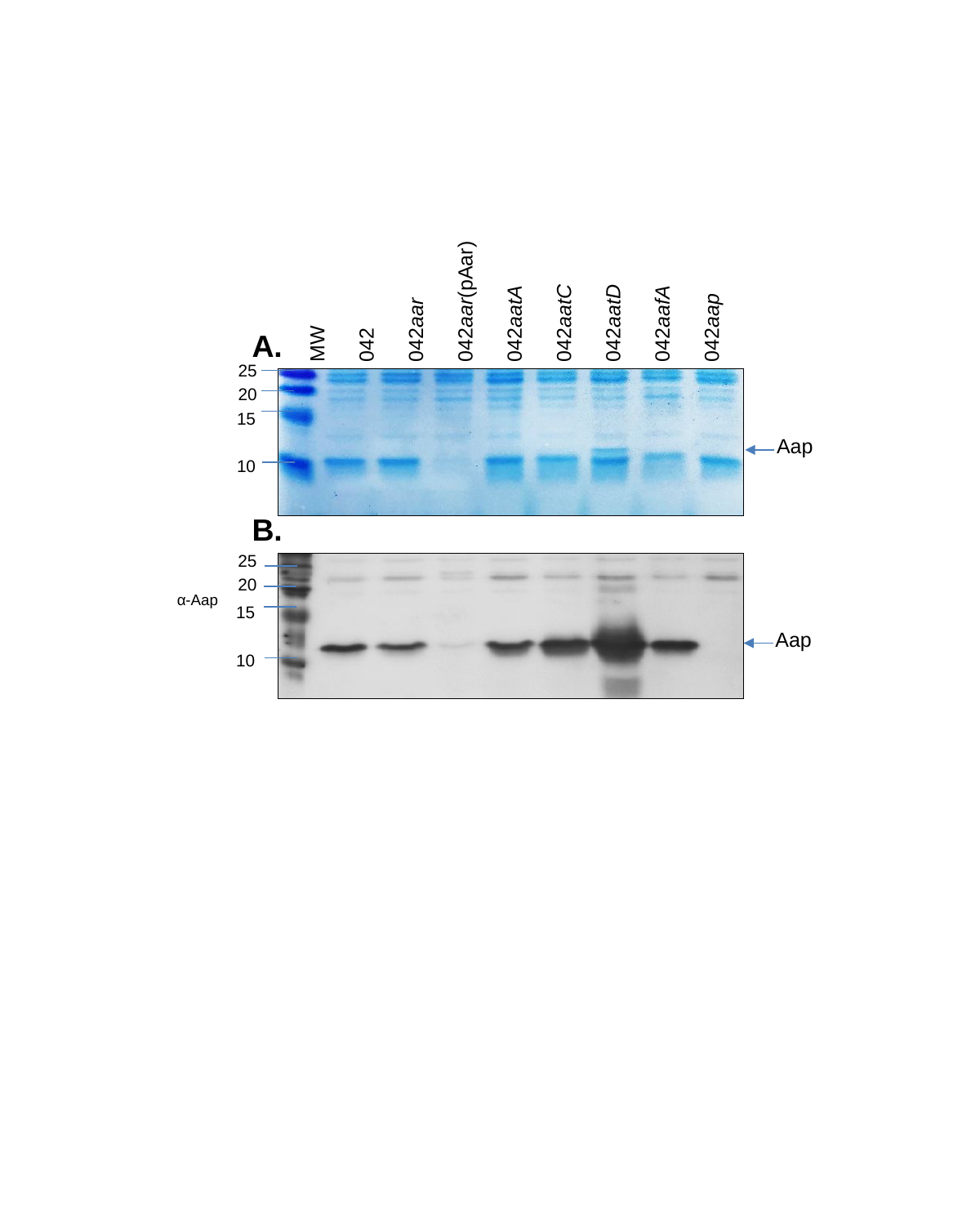

MW
042
042aar
042aar(pAar)
042aatA
042aatC
042aatD
042aafA
042aap
A.
B.
25
20
15
10
25
20
15
10
α-Aap
Aap
Aap

Supplement: S2 Fig — Accumulation of Aap in the periplasmic space of strains containing individual mutations in aatA, aatC, and aatD was evaluated on 20% SDS-PAGE gels (Panel A) and by Western blot with an anti-Aap antibody (Panel B). (PPTX) [file ppat.1008776.s002.pptx]

## Slide 1
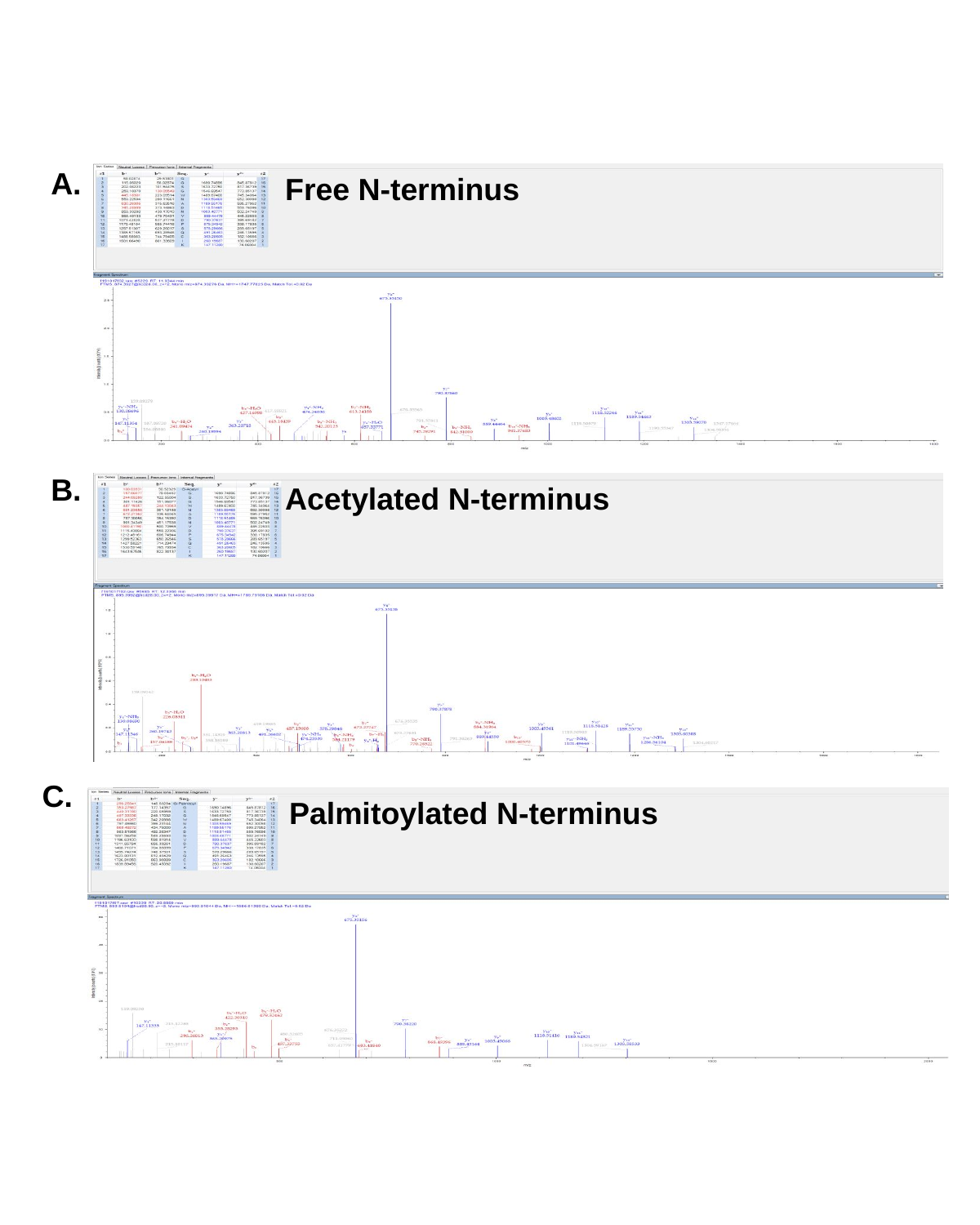

A.
Free N-terminus
B.
Acetylated N-terminus
C.
Palmitoylated N-terminus

Supplement: S4 Fig — Samples were digested with trypsin and post-translational modifications were analyzed. The N-terminal peptide beginning with Gly22 (first 21 amino acids never observed) through Lys38 with a free amino terminus (Panel A). The same peptide with a +42Da mass addition corresponding to an acetylated protein N-terminus (Panel B). Supplementary Panel C shows the peptide with a +238Da mass addition. This peptide is only observed in the 042aap(pAap) sample and corresponds to a protein N-terminal palmitoylation. (PPTX) [file ppat.1008776.s004.pptx]
