# Supplementary material for: Characterization of a novel AraC/XylS-regulated family of N-acyltransferases in pathogens of the order Enterobacterales
Source: PLoS Pathog. 2020 Aug 26;16(8):e1008776. doi: 10.1371/journal.ppat.1008776 (PMC7478709; doi:10.1371/journal.ppat.1008776)
Supplement: S3 Fig — 042 derivatives (042aap, 042aatD aap and 042aatD aatC) were transformed with pAap59-cherry and grown statically in DMEM overnight at 37°C. Bacterial cells were harvested, washed with PBS and incubated with CellBrite stain (green, for membrane staining) and Hoechst 33342 stain (blue, for DNA staining) for 1 h. Bacterial cells were analyzed using a LSM-710 laser-scanning confocal microscope (Zeiss, Germany). Representative confocal images taken with the 64X oil objective are shown (Panel A-T). Of note, close-ups of these figures are shown in Fig 6 of the manuscript. (PPTX) [file ppat.1008776.s003.pptx]

## Slide 1
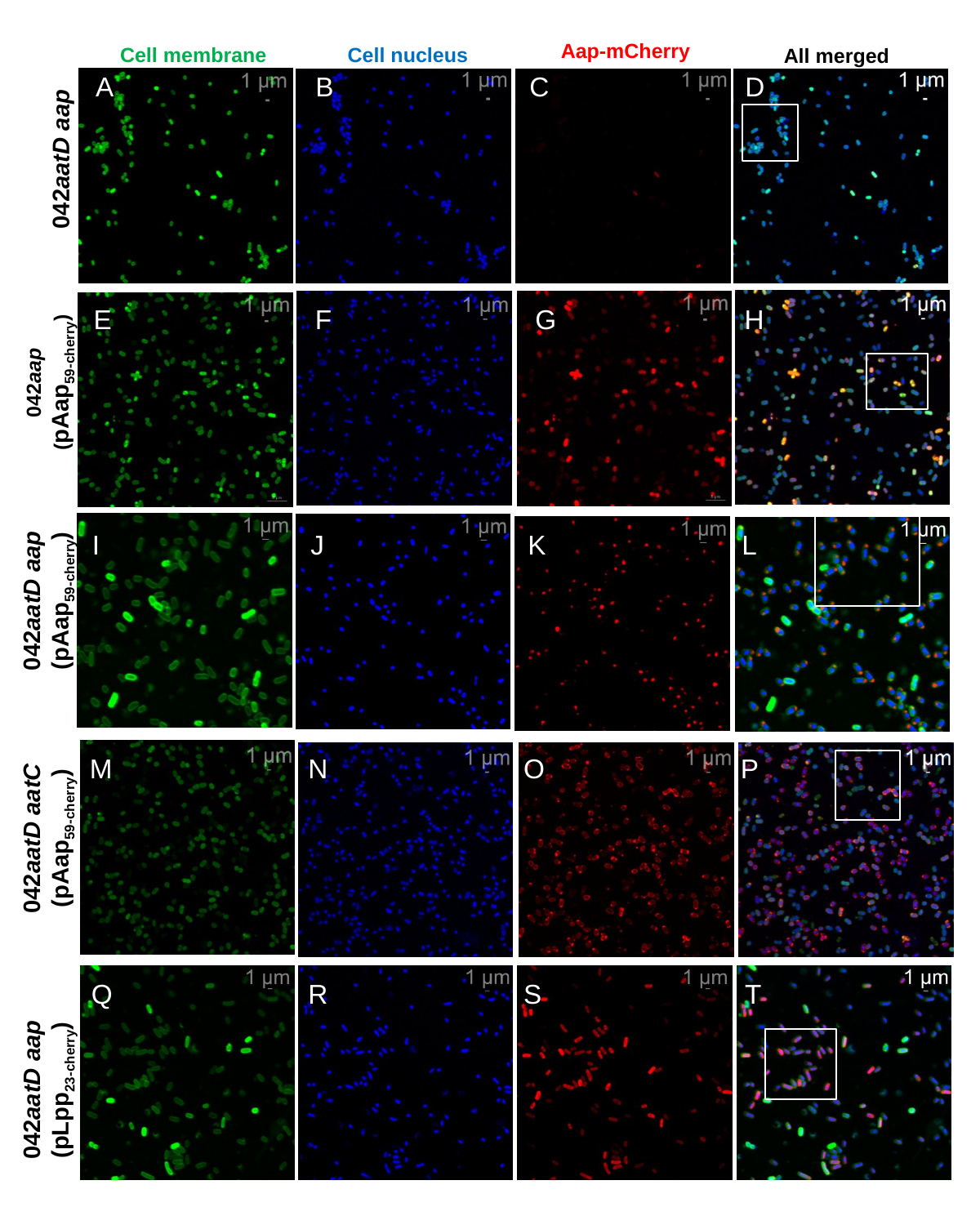

A B C D
Aap-mCherry
Cell membrane
Cell nucleus
All merged
E F G H
I J K L
M N O P
Q R S T
A B C D
042aatD aap
042aap
(pAap59-cherry)
042aatD aap
(pAap59-cherry)
042aatD aatC
(pAap59-cherry)
042aatD aap
(pLpp23-cherry)
