# Supplementary material for: Characterization of a novel AraC/XylS-regulated family of N-acyltransferases in pathogens of the order Enterobacterales
Source: PLoS Pathog. 2020 Aug 26;16(8):e1008776. doi: 10.1371/journal.ppat.1008776 (PMC7478709; doi:10.1371/journal.ppat.1008776)
Supplement: S1 Table — Untargeted metabolomic analysis was performed in 042 and 042aar strains by Liquid chromatography / mass spectrometry (LC/MS). The raw data were acquired and aligned by using the Makerlynx software (version 4.1) based on the m/z value and the retention time of the ion signals. Metabolites were identified in Mass Bank database. Raw data from experiments 1and 2 are listed on Tables 1A and 1B respectively. (DOCX) [file ppat.1008776.s005.docx]

| \| **Table 1A. Metabolomic raw data exp. 1** \| \| \| \| \| \| \| \| \|  \| \| --- \| --- \| --- \| --- \| --- \| --- \| --- \| --- \| --- \| --- \| \| **Ret. Time** \| **m/z** \| **Name** \| **Monoisotopic Mass** \| **042** \| **042** \| **Average** \| **042*aar*** \| **042*aar*** \| **Average** \| \| 26.8318 \| 660.4603 \| PE(14:1(9Z)/16:0) \| 661.4683 \| 0 \| 16.3503 \| 8.17515 \| 565.8607 \| 7.6897 \| 286.7752 \| \| 26.2207 \| 688.4902 \| PE(16:1(9Z)/16:0) \| 689.4996 \| 0 \| 6.4095 \| 3.20475 \| 0 \| 320.7878 \| 160.3939 \| \| 29.2805 \| 662.473 \| PE(14:0/16:0) \| 663.4839 \| 0.507 \| 4.1408 \| 2.3239 \| 257.7289 \| 1.8957 \| 129.8123 \| \| 26.8397 \| 686.4749 \| PE(18:1(11Z)/14:1(9Z)) \| 687.4839 \| 0 \| 2.9718 \| 1.4859 \| 183.7628 \| 1.9015 \| 92.83215 \| \| 26.4704 \| 660.4578 \| PE(14:1(9Z)/16:0) \| 661.4683 \| 0 \| 51.1402 \| 25.5701 \| 131.9825 \| 4.5434 \| 68.26295 \| \| 26.4228 \| 686.4731 \| PE(18:2(9Z,12Z)/14:0) \| 687.4839 \| 1.3014 \| 5.3693 \| 3.33535 \| 156.7999 \| 1.4117 \| 79.1058 \| \| 21.9129 \| 688.4924 \| PE(18:1(9Z)/14:0) \| 689.4996 \| 0 \| 1.05 \| 0.525 \| 0 \| 110.75 \| 55.375 \| \| 25.2699 \| 660.4598 \| PE(14:0/16:1(9Z)) \| 661.4683 \| 0 \| 0 \| 0 \| 79.4725 \| 1.7866 \| 40.62955 \| \| 29.2733 \| 660.4568 \| PE(16:1(9Z)/14:0) \| 661.4683 \| 0 \| 43.2832 \| 21.6416 \| 36.1646 \| 1.5093 \| 18.83695 \| \| 27.2823 \| 688.4906 \| PE(18:0/14:1(9Z)) \| 689.4996 \| 0 \| 0 \| 0 \| 29.0133 \| 45.4446 \| 37.22895 \| \|  \|  \|  \|  \|  \| **Average** \| **6.626175** \|  \|  \| **96.92528** \| \| **Ret. Time** \| **m/z** \| **Name** \| **Monoisotopic Mass** \| **042** \| **042** \| **Average** \| **042*aar*** \| **042*aar*** \| **Average** \| \| 26.8354 \| 728.5255 \| PE-NMe(20:1(11Z)/14:1(9Z)) \| 729.5309 \| 0 \| 0.6868 \| 0.3434 \| 0 \| 2849.3423 \| 1424.671 \| \| 26.2768 \| 702.507 \| PE-NMe(14:1(9Z)/18:0) \| 703.5152 \| 2.9032 \| 6.6168 \| 4.76 \| 0 \| 2531.3411 \| 1265.671 \| \| 26.0337 \| 702.5046 \| PE-NMe(14:1(9Z)/18:0) \| 703.5152 \| 0 \| 0 \| 0 \| 11.463 \| 2079.8293 \| 1045.646 \| \| 10.9183 \| 688.4945 \| PE-NMe(15:0/16:1(9Z)) \| 689.4996 \| 0 \| 0.9855 \| 0.49275 \| 0 \| 842.0306 \| 421.0153 \| \| 25.1732 \| 688.4902 \| PE-NMe(16:1(9Z)/15:0) \| 689.4996 \| 1.1533 \| 15.8041 \| 8.4787 \| 0 \| 507.7829 \| 253.8915 \| \| 26.8165 \| 700.4915 \| PE-NMe(18:1(11Z)/14:1(9Z)) \| 701.4996 \| 0 \| 0 \| 0 \| 419.9444 \| 24.493 \| 222.2187 \| \| 26.7845 \| 674.4757 \| PE-NMe(14:1(9Z)/16:0) \| 675.4839 \| 1.5323 \| 0 \| 0.76615 \| 326.3319 \| 12.8832 \| 169.6076 \| \| 26.2858 \| 660.4596 \| PE-NMe(15:0/14:1(9Z)) \| 661.4683 \| 0 \| 14.938 \| 7.469 \| 234.6294 \| 0 \| 117.3147 \| \| 27.0487 \| 728.5284 \| PE-NMe(16:1(9Z)/18:1(11Z)) \| 729.5309 \| 0 \| 1.0868 \| 0.5434 \| 0.6326 \| 179.8388 \| 90.2357 \| \| 29.2774 \| 700.49 \| PE-NMe(14:0/18:2(9Z,12Z)) \| 701.4996 \| 1.057 \| 19.6648 \| 10.3609 \| 115.5537 \| 2.8194 \| 59.18655 \| \| 26.8067 \| 676.4715 \| PE-NMe(14:1(9Z)/16:0) \| 675.4839 \| 21.2883 \| 64.2411 \| 42.7647 \| 0 \| 29.5434 \| 14.7717 \| \| 28.8382 \| 702.5035 \| PE-NMe(16:0/16:1(9Z)) \| 703.5152 \| 0 \| 0 \| 0 \| 9.0246 \| 92.7759 \| 50.90025 \| \| 29.3027 \| 674.4732 \| PE-NMe(14:1(9Z)/16:0) \| 675.4839 \| 1.2796 \| 0 \| 0.6398 \| 76.676 \| 2.179 \| 39.4275 \| \|  \|  \|  \|  \|  \| **Average** \| **7.66188** \|  \|  \| **398.0429** \| \| **Ret. Time** \| **m/z** \| **Name** \| **Monoisotopic Mass** \| **042** \| **042** \| **Average** \| **042*aar*** \| **042*aar*** \| **Average** \| \| 26.4525 \| 702.5083 \| PE-NMe2(16:1(9Z)/15:0) \| 703.5152 \| 2.0711 \| 0 \| 1.03555 \| 8.2681 \| 8397.6064 \| 4202.937 \| \| 27.1569 \| 702.5056 \| PE-NMe2(16:1(9Z)/15:0) \| 703.5152 \| 0 \| 1.4694 \| 0.7347 \| 0 \| 4486.3032 \| 2243.152 \| \| 29.2965 \| 702.5052 \| PE-NMe2(16:1(9Z)/15:0) \| 703.5152 \| 23.4919 \| 46.8721 \| 35.182 \| 429.3569 \| 3930.5264 \| 2179.942 \| \| 29.2789 \| 688.4884 \| PE-NMe2(16:1(9Z)/14:0) \| 689.4996 \| 80.7174 \| 140.0825 \| 110.39995 \| 3537.563 \| 0 \| 1768.782 \| \| 26.033 \| 688.4892 \| PE-NMe2(16:1(9Z)/14:0) \| 689.4996 \| 0 \| 4.5795 \| 2.28975 \| 21.0948 \| 3257.4739 \| 1639.284 \| \| 26.8368 \| 688.4938 \| PE-NMe2(16:0/14:1(9Z)) \| 689.4996 \| 0 \| 0 \| 0 \| 446.1473 \| 2521.5164 \| 1483.832 \| \| 26.682 \| 702.5098 \| PE-NMe2(15:0/16:1(9Z)) \| 703.5152 \| 0 \| 11.8139 \| 5.90695 \| 0 \| 2388.425 \| 1194.213 \| \| 29.4748 \| 702.5082 \| PE-NMe2(16:1(9Z)/15:0) \| 703.5152 \| 0 \| 7.2924 \| 3.6462 \| 17.3932 \| 1765.3755 \| 891.3844 \| \| 26.4594 \| 714.5088 \| PE-NMe2(18:2(9Z,12Z)/14:0) \| 715.5152 \| 2.3222 \| 3.5208 \| 2.9215 \| 70.0653 \| 1269.5612 \| 669.8133 \| \| 29.2784 \| 714.5043 \| PE-NMe2(14:1(9Z)/18:1(11Z)) \| 715.5152 \| 59.9396 \| 55.5243 \| 57.73195 \| 942.2031 \| 16.7867 \| 479.4949 \| \| 26.0408 \| 714.505 \| PE-NMe2(18:2(9Z,12Z)/14:0) \| 715.5152 \| 1.9127 \| 10.6602 \| 6.28645 \| 15.0104 \| 964.7491 \| 489.8798 \| \| 26.8286 \| 714.5103 \| PE-NMe2(16:1(9Z)/16:1(9Z)) \| 715.5152 \| 2.7432 \| 1.8706 \| 2.3069 \| 164.6452 \| 787.8879 \| 476.2666 \| \| 22.9423 \| 688.4914 \| PE-NMe2(16:1(9Z)/14:0) \| 689.4996 \| 0 \| 0.7294 \| 0.3647 \| 0 \| 708.2003 \| 354.1002 \| \| 22.5954 \| 688.4884 \| PE-NMe2(14:0/16:1(9Z)) \| 689.4996 \| 0.5814 \| 1.4582 \| 1.0198 \| 0 \| 683.2193 \| 341.6097 \| \| 24.3557 \| 702.5045 \| PE-NMe2(15:0/16:1(9Z)) \| 703.5152 \| 1.6913 \| 3.8827 \| 2.787 \| 1.4762 \| 325.3788 \| 163.4275 \| \| 23.9749 \| 688.4951 \| PE-NMe2(14:0/16:1(9Z)) \| 689.4996 \| 0 \| 2.0551 \| 1.02755 \| 0 \| 271.8589 \| 135.9295 \| \| 26.6375 \| 660.4593 \| PE-NMe2(14:1(9Z)/14:0) \| 661.4683 \| 0 \| 34.255 \| 17.1275 \| 90.5319 \| 0 \| 45.26595 \| \|  \|  \|  \|  \|  \| **Average** \| **15.6730281** \|  \|  \| **1103.489** \| \| **LysoPE** \| **m/z** \| **Name** \| **Monoisotopic Mass** \| **042** \| **042** \| **Average** \| **042*aar*** \| **042*aar*** \| **Average** \| \| 22.7256 \| 450.2612 \| LysoPE(0:0/16:1(9Z)) \| 451.2699 \| 9431.2529 \| 5212.5596 \| 7321.90625 \| 9028.3398 \| 15706.5469 \| 12367.44 \| \| 24.1799 \| 478.2933 \| LysoPE(0:0/18:1(9Z)) \| 479.3012 \| 5316.3257 \| 2331.2715 \| 3823.7986 \| 4169.5942 \| 8372.0771 \| 6270.836 \| \| 23.9061 \| 452.2777 \| LysoPE(0:0/16:0) \| 453.2855 \| 3780.7688 \| 2309.6052 \| 3045.187 \| 4745.96 \| 5038.2046 \| 4892.082 \| \| 22.3554 \| 450.261 \| LysoPE(0:0/16:1(9Z)) \| 451.2699 \| 2151.1125 \| 1988.2753 \| 2069.6939 \| 2106.6423 \| 4083.7891 \| 3095.216 \| \| 22.1466 \| 424.2465 \| LysoPE(0:0/14:0) \| 425.2542 \| 740.045 \| 261.1004 \| 500.5727 \| 799.4543 \| 1688.4697 \| 1243.962 \| \| 23.9299 \| 478.292 \| LysoPE(18:1(9Z)/0:0) \| 479.3012 \| 0.4283 \| 1002.0968 \| 501.26255 \| 915.9527 \| 1526.9044 \| 1221.429 \| \| 20.5392 \| 422.2278 \| LysoPE(14:1(9Z)/0:0) \| 423.2386 \| 148.3658 \| 41.2056 \| 94.7857 \| 182.286 \| 612.6563 \| 397.4712 \| \| 21.6979 \| 424.2466 \| LysoPE(14:0/0:0) \| 425.2542 \| 120.8617 \| 32.4454 \| 76.65355 \| 21.0867 \| 272.9622 \| 147.0245 \| \| 24.3402 \| 478.2945 \| LysoPE(0:0/18:1(11Z)) \| 479.3012 \| 94.9146 \| 11.3869 \| 53.15075 \| 4.1361 \| 183.3463 \| 93.7412 \| \| 23.1311 \| 438.2636 \| LysoPE(0:0/15:0) \| 439.2699 \| 40.3112 \| 6.2968 \| 23.304 \| 68.9513 \| 115.6255 \| 92.2884 \| \| 25.156 \| 480.308 \| LysoPE(18:0/0:0) \| 481.3168 \| 17.0893 \| 59.2802 \| 38.18475 \| 58.7123 \| 48.1831 \| 53.4477 \| \|  \|  \|  \|  \|  \| **Geomean** \| **364.775066** \|  \|  \| **759.0806** \| |
| --- | --- | --- | --- | --- | --- | --- | --- | --- | --- | --- | --- | --- | --- | --- | --- | --- | --- | --- | --- | --- | --- | --- | --- | --- | --- | --- | --- | --- | --- | --- | --- | --- | --- | --- | --- | --- | --- | --- | --- | --- | --- | --- | --- | --- | --- | --- | --- | --- | --- | --- | --- | --- | --- | --- | --- | --- | --- | --- | --- | --- | --- | --- | --- | --- | --- | --- | --- | --- | --- | --- | --- | --- | --- | --- | --- | --- | --- | --- | --- | --- | --- | --- | --- | --- | --- | --- | --- | --- | --- | --- | --- | --- | --- | --- | --- | --- | --- | --- | --- | --- | --- | --- | --- | --- | --- | --- | --- | --- | --- | --- | --- | --- | --- | --- | --- | --- | --- | --- | --- | --- | --- | --- | --- | --- | --- | --- | --- | --- | --- | --- | --- | --- | --- | --- | --- | --- | --- | --- | --- | --- | --- | --- | --- | --- | --- | --- | --- | --- | --- | --- | --- | --- | --- | --- | --- | --- | --- | --- | --- | --- | --- | --- | --- | --- | --- | --- | --- | --- | --- | --- | --- | --- | --- | --- | --- | --- | --- | --- | --- | --- | --- | --- | --- | --- | --- | --- | --- | --- | --- | --- | --- | --- | --- | --- | --- | --- | --- | --- | --- | --- | --- | --- | --- | --- | --- | --- | --- | --- | --- | --- | --- | --- | --- | --- | --- | --- | --- | --- | --- | --- | --- | --- | --- | --- | --- | --- | --- | --- | --- | --- | --- | --- | --- | --- | --- | --- | --- | --- | --- | --- | --- | --- | --- | --- | --- | --- | --- | --- | --- | --- | --- | --- | --- | --- | --- | --- | --- | --- | --- | --- | --- | --- | --- | --- | --- | --- | --- | --- | --- | --- | --- | --- | --- | --- | --- | --- | --- | --- | --- | --- | --- | --- | --- | --- | --- | --- | --- | --- | --- | --- | --- | --- | --- | --- | --- | --- | --- | --- | --- | --- | --- | --- | --- | --- | --- | --- | --- | --- | --- | --- | --- | --- | --- | --- | --- | --- | --- | --- | --- | --- | --- | --- | --- | --- | --- | --- | --- | --- | --- | --- | --- | --- | --- | --- | --- | --- | --- | --- | --- | --- | --- | --- | --- | --- | --- | --- | --- | --- | --- | --- | --- | --- | --- | --- | --- | --- | --- | --- | --- | --- | --- | --- | --- | --- | --- | --- | --- | --- | --- | --- | --- | --- | --- | --- | --- | --- | --- | --- | --- | --- | --- | --- | --- | --- | --- | --- | --- | --- | --- | --- | --- | --- | --- | --- | --- | --- | --- | --- | --- | --- | --- | --- | --- | --- | --- | --- | --- | --- | --- | --- | --- | --- | --- | --- | --- | --- | --- | --- | --- | --- | --- | --- | --- | --- | --- | --- | --- | --- | --- | --- | --- | --- | --- | --- | --- | --- | --- | --- | --- | --- | --- | --- | --- | --- | --- | --- | --- | --- | --- | --- | --- | --- | --- | --- | --- | --- | --- | --- | --- | --- | --- | --- | --- | --- | --- | --- | --- | --- | --- | --- | --- | --- | --- | --- | --- | --- | --- | --- | --- | --- | --- | --- | --- | --- | --- | --- | --- | --- | --- | --- | --- | --- | --- | --- | --- | --- | --- | --- | --- | --- | --- | --- | --- | --- | --- | --- | --- | --- | --- | --- | --- | --- | --- | --- | --- | --- | --- | --- | --- | --- | --- | --- | --- | --- | --- | --- | --- | --- | --- | --- | --- | --- | --- | --- | --- | --- | --- | --- | --- | --- | --- | --- | --- | --- | --- | --- | --- | --- | --- | --- | --- | --- | --- | --- | --- | --- | --- | --- | --- | --- | --- | --- | --- | --- | --- | --- | --- | --- | --- | --- | --- | --- | --- | --- | --- | --- | --- | --- | --- | --- | --- | --- | --- | --- | --- | --- | --- | --- | --- | --- | --- | --- | --- | --- | --- | --- | --- | --- | --- | --- |

| **Table 1B. Metabolomic raw data exp. 2** | | | | | |  |
| --- | --- | --- | --- | --- | --- | --- |
| **LysoPE** | **042** | **042** | **Average** | **042*aar*** | **042*aar*** | **Average** |
| LysoPC(15:0) | 6.7685 | 2.9711 | 4.8698 | 6.8043 | 7.6987 | 7.2515 |
| LysoPE(0:0/18:1(9Z)) | 158.0633 | 175.613 | 166.83815 | 217.8098 | 255.2513 | 236.53055 |
| LysoPC(14:0/0:0) | 3.0767 | 2.6651 | 2.8709 | 3.2834 | 1.6072 | 2.4453 |
| LysoPC(14:1(9Z)) | 193.6395 | 227.9112 | 210.77535 | 399.2666 | 425.4621 | 412.36435 |
| LysoPE(0:0/16:0) | 248.9805 | 208.6568 | 228.81865 | 212.4173 | 160.9762 | 186.69675 |
| LysoPE(0:0/16:1(9Z)) | 171.005 | 212.4305 | 191.71775 | 251.0492 | 404.0645 | 327.55685 |
| LysoPE(16:1(9Z)/0:0) | 1.4786 | 1.4299 | 1.45425 | 1.0531 | 5.0463 | 3.0497 |
| LysoPE(0:0/15:0) | 6.4859 | 7.214 | 6.84995 | 7.0533 | 16.8917 | 11.9725 |
| LysoPE(0:0/14:0) | 14.4586 | 16.5417 | 15.50015 | 27.6765 | 38.4935 | 33.085 |
| LysoPE(0:0/14:1(9Z)) | 1.4563 | 1.8815 | 1.6689 | 4.6601 | 10.5564 | 7.60825 |
| LysoPE(14:1(9Z)/0:0) | 0.5423 | 1.2774 | 0.90985 | 2.7334 | 9.4411 | 6.08725 |
