# Supplementary material for: Characterization of a novel AraC/XylS-regulated family of N-acyltransferases in pathogens of the order Enterobacterales
Source: PLoS Pathog. 2020 Aug 26;16(8):e1008776. doi: 10.1371/journal.ppat.1008776 (PMC7478709; doi:10.1371/journal.ppat.1008776)
Supplement: S2 Table — (DOCX) [file ppat.1008776.s006.docx]

| **Supplementary Table 2. Strains used in this study** | | |
| --- | --- | --- |
| **Name** | **Characteristics** | **Source** |
| EAEC 042 | Enteroaggregative *E. coli* strain 042 | Lab collection |
| 042*aar* | 042 mutant in *aar* created by lambda red technique | Santiago et al. 2014 |
| 042*aar*(pAar) | 042 mutant in *aar* complemented in trans with pAar | Santiago et al. 2014 |
| 042*aafA* | 042 mutant in *aafA* created by transposon procedures | Lab collection |
| 042*aap-λ* | 042 mutant in *aap* created by lambda red technique | This study |
| 042*aap-T* | 042 mutant in *aap* created by transposon procedures | Lab collection |
| 042*aatD-λ* | 042 mutant in *aatD* created by lambda red technique | This study |
| 042*aatD-T* | 042 mutant in *aatD* created by transposon procedures | Lab collection |
| 042*aatD aap* | 042 mutant in *aatD and aap* created by lambda red technique. Km marker is inserted in *aap* gene | This study |
| 042*aap aatD* | 042 mutant in *aap and aatD* created by lambda red technique. Km marker is inserted in *aatD* gene | This study |
| 042*aatD*(pAatD_EAEC_) | 042 mutant in *aatD* created by lambda red technique and complemented in trans with AatD from EAEC | This study |
| 042*aatD*(pAatD_E207A_) | 042 mutant in *aatD* created by lambda red technique and complemented in trans with pAatD_E207A_ derivative | This study |
| 042*aatD*(pAatD_C316A_) | 042 mutant in *aatD* created by lambda red technique and complemented in trans with pAatD_C316A_ derivative | This study |
| 042*aatD*(pLnt_EAEC_) | 042 mutant in *aatD* created by lambda red technique and complemented in trans with Lnt from EAEC | This study |
| 042*aatD*(pAatD_ETEC_) | 042 mutant in *aatD* created by lambda red technique and complemented in trans with AatD from ETEC | This study |
| 042*aatD*(pAatD*_Cr_)* | 042 mutant in *aatD* created by lambda red technique and complemented in trans with AatD from *C. rodentium* | This study |
| 042*aatD aap*(pAap_042_) | 042 mutant in *aatD* and *aap* created by lambda red technique and complemented in trans with Aap from EAEC | This study |
| 042*aatD aap*(pCexE) | 042 mutant in *aatD* and *aap* created by lambda red technique and complemented in trans with CexE | This study |
| 042*aatD aap*(pAap_042-H6_) | 042 mutant in *aatD* and *aap* strain transformed with pAap_042-H6_. Used for purification of Aap. | This study |
| 042*aap*(pAap_042-H6_) | 042 mutant in *aap* strain transformed with pAap_042-H6_. Used for purification of Aap. | This study |
| 042*aap*(pAap_59-Cherry_) | 042 mutant in *aap* strain transformed with pAap_59-Cherry_. Used in the confocal microscopy experiments. | This study |
| 042*aatD aap*(pAap_59-Cherry_) | 042 mutant in *aap* and *aatD* strain transformed with pAap_59-Cherry_. Used in the confocal microscopy experiments. | This study |
| 042*aatD aatC*(pAap_59-Cherry_) | 042 mutant in *aatD* and *aatC* strain transformed with pAap_59-Cherry_. Used in the confocal microscopy experiments. | This study |
| 042*aatD aap*(pLpp_23-Cherry_) | 042 mutant in *aap* and *aatD* strain transformed with pLpp_23-Cherry_. Used in the confocal microscopy experiments. | This study |
