# Supplementary material for: Characterization of a novel AraC/XylS-regulated family of N-acyltransferases in pathogens of the order Enterobacterales
Source: PLoS Pathog. 2020 Aug 26;16(8):e1008776. doi: 10.1371/journal.ppat.1008776 (PMC7478709; doi:10.1371/journal.ppat.1008776)
Supplement: S3 Table — (DOCX) [file ppat.1008776.s007.docx]

| **Supplementary Table 3. Primers used in this study** | |
| --- | --- |
| **Name** | **DNA Sequences (5→3)** |
| **Primers for pBAD derivatives (NdeI/XbaI)** | |
| AatD-F_EAEC_ | CCGTAATCATATGAAATTCGCTATTGTCTTATTGTA |
| AatD-R_EAEC_ | CCGTAATTCTAGATTAGTGGTGGTGGTGGTGGTGTATCTGTGTAAATAAAAAAGGTTCCG |
| AatD-F_ETEC_ | CCGTAATCATATGATTGTTAAAATGGAGAATATAATTGCAT |
| AatD-R_ETEC_ | CCGTAATTCTAGATTAGTGGTGGTGGTGGTGGTGTACATCAATGACAAAAAATGG |
| AatD-F_Cr_ | CCGTAATCATATGATAGAAGCGTTCATTCCAACATTGTCTCTT |
| AatD-R_Cr_ | CCGTAATTCTAGATTAGTGGTGGTGGTGGTGGTGAATTTCCAAAAGGAATATTGCTTG |
| Lnt-F_EAEC_ | CGTATCGTCATATGGCTTTTGCCTCATTAATTGAACGCCAGCGCATTC |
| Lnt-R_EAEC_ | CGTATCGTTCTAGATTAACGTTTACGTGCTCGCAGACTCATCAACAC |
| Aap-F_042_ | CCGTAATCATATGAAAAAAATTAAGTTTGTTATCTTTTCTGGC |
| Aap-R_042_ | CCGTAATTCTAGATTATTTAACCCATTCGGTTAGAGCACGATATTTTTG |
| Aap-R_042-H6_ | CGTAATTCTAGATTAGTGGTGGTGGTGGTGGTGTTTAACCCATTCGGTTAGAGCACGATA |
| CexE-F_ETEC_ | CCGTAATCATATGAAAAAATATATATTAGGTGTTATTCTGGCTATGGGG |
| CexE-R_ETEC_ | CCGTAATTCTAGATTATTTATACCAATAAGGGGTGTCACCACCTGCAGATACTTT |
| **Primers employed for confocal microscopy constructs** | |
| Aap-S_59-cherry_ | GCTATGCTCTAGAACTCATATATACTTTAGATTGATTTACGCGCCCTGTA |
| Aap-R_59-cherry_ | GCTATGCAGATCTTTCATTAAGGCCTTGCATACATACTGAGACACCGCT |
| Cherry-S | GCTATGCAGATCTGTGAGCAAGGGCGAGGAGGATAACATGGC |
| Cherry-R | GCTATGCGCTAGCGTATATATGAGTAAACTTGGTCTGACAG |
| Lpp-S_23-cherry_ | GCTATGCCATATGAAAGCTACTAAACTGGTACTGGGCGCGGTA |
| Lpp-R_23-cherry_ | GCTATGCAGATCTGCTGGAGCAACCTGCCAGCAGAGTAGAACCTAG |
| **Primers employed for lambda red procedures** | |
| Aap_042_ Forward | TCTGGCCGCAACTCTTATTTATGCTAGCCTTCTAAAAGGAGGGGCGGCATTGGCTGAATTATAACCTCTAAATATCGTAATTATTTATTGTGAAAAATACCTCTATATACATGGGGAATATCTAGAGAGAAGTCATATGAAAAAAATTAAGTTTGTTATCTTTTCTGGCATCTTGGGTATgtgtaggctggagctgcttc |
| Aap_042_ Reverse | TGGTGGTCTCAGCCCGGAACAATTTGAAAACCAGAACCTCGCTTAGACCTGTGTCCATATTACGAGGGTGGGATCAATCATAAAAAATGTCATTCCCAAAACGCTGGATAGGAATCCAGCGTGGAGAGTTGAAATTTTAGCTAGAGCTAGATATTATTTAACCCATTCGGTTAGAGCACGATGGGAATTAGCCATGGTCC |
| AatC_EAEC_ Forward | ATGATTAGAGTAAAAATACATAAAAAACCTATAGAAAACAGAACTATCCTGAATAATAGCACTATTGAGATAAAAGAGGGATCGTTCAATATTATTACTGGCCCGTCTGGAGTTGGAAAGACTTCACTGCTTAACATTATTGGTCTATTAGATAATGCCTTTGTTGGAGAGTATGAACTTgtgtaggctggagctgcttc |
| AatC_EAEC_ Reverse | GTAAGCCTATGTATTTAATAGTTGGATTAAAGTCATATTCTCATCGATTAGTTCAAGATCATGAGTTACCATAACGACTGTTCCTCCTTGATTATTGTATTCAGATAGTATATTCATCACTAATTTTTTATTCTTTATATCTAGACTAGCAGTAGGTTCATCTGCTAAAAGTATACTGGGATGGGAATTAGCCATGGTCC |
| AatD_EAEC_ Forward | CTCATGATCTTGAACTAATCGATGAGAATATGACTTTAATCCAACTATTAAATACATAGGCTTACAGTTTATGAAATTCGCTATTGTCTTATTGTATTTTTTTGCCTATTATCTTGCAGCAAGAAAAAGACGAGTGAGTCTTTTTTTTACTATTCTTTTATACTCTATCATTTTCTCTGGgtgtaggctggagctgcttc |
| AatD_EAEC_ Reverse | ATACATTAGTTAATATAATAGCACTTCTATTTTCAACTGAAAGTTAGTGCATTACTCATATCTGTGTAAATAAAAAAGGTTCCGTTTTTGACCTATTAAATAAGTCCATATCAATTTTCCAATTATTGTCTATAAAAACTGTCCCACCATAATTTTGAATATTAATAAGAGGAGTGTTCGATGGGAATTAGCCATGGTCC |
| **Primers employed for site mutations in AatD** | |
| 207EAsen | GAAGGTTGATCTAATTATTCTCTCAGCAAATGTTTTTTTCGGTTACAAGAATG |
| 207EArev | CATTCTTGTAACCGAAAAAAACATTTGCTGAGAGAATAATTAGATCAACCTTC |
| 268KAsen | gcataaagaagaatttcttctccaccaagcaagcaaattgatacctttttttgaga |
| 268KArev | tctcaaaaaaaggtatcaatttgcttgcttggtggagaagaaattcttctttatgc |
| 316CAsen | TGGATTTCAACAACATTAAAATGAGTATTCATATAGCTTATGAGGGATTATTCCCTG |
| 316CArev | CAGGGAATAATCCCTCATAAGCTATATGAATACTCATTTTAATGTTGTTGAAATCCA |
